# Detection of white matter microstructural changes in patients with systemic lupus erythematosus based on multiple diffusion models and related diffusion metrics

**DOI:** 10.4103/NRR.NRR-D-25-00730

**Published:** 2025-11-25

**Authors:** Zhenxing Li, Huanhuan Li, Bailing Tian, Huiyang Liu, Yueluan Jiang, Pingting Yang, Guoguang Fan, Hu Liu

**Affiliations:** 1 Department of Radiology, The First Hospital of China Medical University, Shenyang, Liaoning Province, China; 2 Department of Rheumatology and Immunology, The First Hospital of China Medical University, Shenyang, Liaoning Province, China; 3 MR Research Collaboration Team, Siemens Healthineers Co., Ltd., Beijing, China

**Keywords:** diffusion kurtosis imaging, diffusion tensor imaging, mean apparent propagator, neurite orientation dispersion and density imaging, neuropsychiatric systemic lupus erythematosus, return to axis probability, return to origin probability, superior longitudinal fasciculus-3, superior thalamic radiation, tract-based spatial statistics, white matter microstructure

## Abstract

Some patients with systemic lupus erythematosus experience neuropsychiatric symptoms. Although magnetic resonance imaging can detect abnormal signals in the white matter of the brain, conventional methods often struggle to accurately capture microstructural changes. Various diffusion models have been used to study white matter in systemic lupus erythematosus; however, comparative analyses of their sensitivity and specificity for detecting microstructural changes remain insufficient. To address this, our team designed a diagnostic trial that used multimodal diffusion imaging techniques to observe white matter microstructural changes in patients with systemic lupus erythematosus who had neuropsychiatric symptoms, with an aim to identify key diagnostic biomarkers for these patients. Patients with active lupus who received treatment at the Department of Rheumatology and Immunology, The First Affiliated Hospital of China Medical University, from September 2023 to March 2024 were recruited. According to the standards of the American College of Rheumatology, patients with systemic lupus erythematosus who had neuropsychiatric symptoms were assigned to the systemic lupus erythematosus group, whereas those without neuropsychiatric symptoms were assigned to the non-systemic lupus erythematosus group. Additionally, healthy volunteers matched by region, sex, and age were recruited as controls. All three groups underwent the same diffusion magnetic resonance imaging examination protocol to compare differences in diffusion parameters. Advanced diffusion imaging models were able to sensitively detect microstructural changes in the white matter fibers of patients with systemic lupus erythematosus who had neuropsychiatric symptoms, with specific diffusion parameters showing significant abnormalities in key brain regions. In the left superior longitudinal fasciculus subregion and the right thalamic radiations of patients with systemic lupus erythematosus who had neuropsychiatric symptoms, we also identified abnormal diffusion characteristics that were clearly correlated with disease activity, suggesting that microstructural changes in these areas may reflect the dynamic process of neuroinflammatory damage. The present study addresses critical challenges in the diagnosis of systemic lupus erythematosus by identifying specific white matter imaging biomarkers and elucidating the association between microstructural damage and clinical manifestations. The main contributions of our study include: 1) establishing axial regression probability parameters from mean apparent propagator magnetic resonance imaging as sensitive biomarkers for systemic lupus erythematosus, particularly in the third subregion of the left superior longitudinal fasciculus; 2) demonstrating that multimodal diffusion imaging may be superior to conventional diffusion tensor imaging for detecting white matter microstructural abnormalities in patients with systemic lupus erythematosus; and 3) integrating tract-based spatial statistics with clinically relevant analyses to link imaging findings to pathological mechanisms.

## Graphical abstract

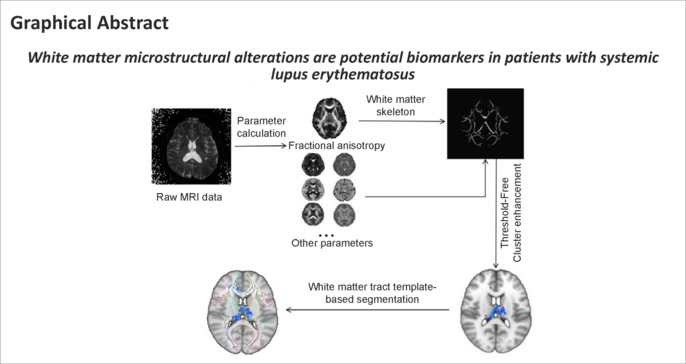

## Introduction

Systemic lupus erythematosus (SLE) is a multisystem autoimmune disorder that predominantly affects young women (Justiz Vaillant et al., 2024). Neuropsychiatric SLE (NPSLE) occurs in 39%–50% of patients, increases morbidity and mortality (Hanly et al., 2019; Sarwar et al., 2021), and manifests as a diverse range of central and peripheral nervous system symptoms (Committee, 1999). Two pathological mechanisms of NPSLE have been proposed: ischemic and autoimmune pathways (Ota et al., 2022), both of which ultimately cause neuronal and glial damage, thus indicating that neural injury plays a critical role in SLE progression. In addition, magnetic resonance imaging (MRI) frequently reveals white matter hyperintensities (WMH) in patients (Papadaki et al., 2018; Inglese et al., 2022), indicating that white matter alterations represent important manifestations of neural injury. Furthermore, compared with non-NPSLE patients, those with NPSLE exhibit a larger volume and more complex shape of periventricular WMH (Inglese et al., 2022). Investigating white matter in SLE and analyzing the patterns of neural fiber damage may provide novel insights into neuroinjury, repair, and regeneration research. A detailed characterization of white matter microstructural integrity is crucial for identifying potential obstacles to neural regeneration and developing targeted repair strategies for SLE patients.

Although studies into white matter microstructural changes in SLE have progressed, several controversies and limitations persist. Diffusion tensor imaging (DTI) remains the primary technique for assessing white matter microstructure in patients with SLE. DTI characterizes Gaussian water diffusion in tissue to infer microstructural properties (Beaulieu, 2002). Previous studies (Correa et al., 2016, 2018) have documented integrity impairments in the thalamic radiations, inferior fronto-occipital fasciculi, superior longitudinal fasciculi, and corpus callosum in patients with SLE.

Research highlights

• Changes in the upper longitudinal fasciculus reflect neurological inflammatory damage in patients with systemic lupus erythematosus.

• Multimodal diffusion imaging can distinguish between neuropsychiatric and non-neuropsychiatric white matter lesions in patients with systemic lupus erythematosus.

• Mean apparent propagator imaging has the highest detection sensitivity for white matter lesions in systemic lupus erythematosus.

• Axial regression probability parameters of the left upper longitudinal fasciculus may serve as biomarkers for systemic lupus erythematosus.

• White matter damage is associated with disease activity, revealing the correlation between systemic inflammation and central nervous system inflammation.

Diffusion kurtosis imaging (DKI), an extension of DTI, accounts for non-Gaussian diffusion effects, thereby providing more comprehensive microstructural information (Jensen and Helpern, 2010; Masutani, 2022; Minosse et al., 2023). However, its application in SLE remains limited, and its advantages for the assessment of neurodamage require further validation. Neurite orientation dispersion and density imaging (NODDI) quantifies neurite density and orientation dispersion by modeling water diffusion across intracellular, extracellular, and cerebrospinal fluid compartments (Schneider et al., 2017; Kamiya et al., 2020). Although reduced neurite density has been reported in patients with non-NPSLE (Hu et al., 2024b), NPSLE-specific fiber alterations remain unexplored. Mean apparent propagator MRI (MAP-MRI) is an advanced diffusion technique that directly measures the probability density function of water molecule spin displacements without making assumptions about diffusion properties (Avram et al., 2022). It has been clinically applied in diffuse gliomas, amyotrophic lateral sclerosis, and Alzheimer’s disease (Chen et al., 2021; Moody et al., 2022; Wang et al., 2023b). Nevertheless, no studies have used MAP-MRI to evaluate white matter changes in NPSLE. Its ability to characterize tissue microstructure at a sophisticated level offers unique opportunities to both monitor neurorestorative processes and evaluate the efficacy of interventions aimed at promoting neural regeneration.

Although multiple diffusion models have been used in SLE white matter research, comparative analyses of their sensitivity and specificity for detecting microstructural alterations remain insufficient. Current studies have predominantly focused on non-NPSLE patients, and comparative investigations of white matter differences between NPSLE and non-NPSLE cohorts are lacking, thus hindering a comprehensive understanding of the mechanisms of neurological involvement in SLE. Understanding these differential patterns is essential for developing targeted neuroregenerative strategies tailored to SLE.

The present study aimed to: (1) systematically analyze white matter microstructural alterations in SLE patients using tract-based spatial statistics (TBSS), using parameters from DTI, DKI, NODDI, and MAP-MRI to identify the most sensitive diffusion model; (2) delineate specific white matter tract differences in patients with SLE, characterizing the most discriminative parameters and fiber bundle regions as potential NPSLE biomarkers; and (3) evaluate the diagnostic efficacy of diffusion parameters using receiver operating characteristic (ROC) curve analysis. Beyond these immediate goals, our findings provide a foundation for developing imaging biomarkers to monitor neural repair processes and assess the effectiveness of future regenerative therapies in patients with SLE.

## Methods

### Participants

**Figure 1:**
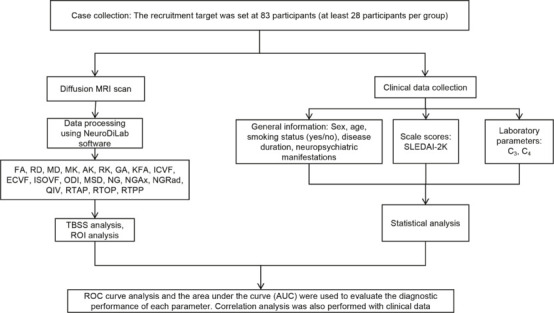
Study design flowchart. AK: Axial kurtosis; ECVF: extracellular volume fraction; FA: fractional anisotropy; GA: geodesic anisotropy; ICVF: intracellular volume fraction; ISOVF: isotropic volume fraction; KFA: kurtosis fractional anisotropy; MD: mean diffusivity; MK: mean kurtosis; MSD: mean squared displacement; NG: non-Gaussianity; NGAx: non-Gaussianity axial; NGRad: non-Gaussianity radial; ODI: orientation dispersion index; QIV: quantitative isotropic volume; RD: radial diffusivity; RK: radial kurtosis; ROC: receiver operating characteristic; ROI: region of interest; RTAP: return-to-axis probability; RTOP: return-to-origin probability; RTPP: return-to-plane probability; TBSS: tract-based spatial statistics.

The present diagnostic study was conducted at The First Hospital of China Medical University (Shenyang, Liaoning Province, China), between September 2023 and March 2024. The study was approved by the Ethics Committee for Medical Research, The First Affiliated Hospital of China Medical University, China (approval No. [2025] 89, approval date: March 7, 2025), and all data were collected prospectively. Written informed consent was obtained from all participants (or their legal guardians for minors) as detailed in the consent form. All procedures performed in the study were conducted in accordance with the ethical standards given in the 1964 *Declaration of Helsinki*, as revised in 2013. The present report was written in accordance with the Standards for Reporting of Diagnostic Accuracy Studies (STARD) 2015 (Bossuyt et al., 2015). The study design flowchart is shown in **Figure 1**.

#### Recruitment

Hospitalized patients with active lupus were recruited from the Department of Rheumatology and Immunology at The First Affiliated Hospital of China Medical University. Healthy volunteers were recruited through the hospital’s health examination center. Patient medical records were collected via the picture archiving and communication system.

#### Inclusion and exclusion criteria

The inclusion criteria were as follows: (1) Aged between 14 and 65 years. (2) The NPSLE group was identified based on the American College of Rheumatology (ACR) diagnostic criteria for NPSLE (Committee, 1999). (3) The non-NPSLE group comprised individuals who met the ACR criteria for SLE without neuropsychiatric manifestations (Hochberg, 1997); all subjects also underwent a comprehensive neurological and neuropsychological evaluation by a rheumatology specialist to ensure appropriate group allocation. (4) The healthy control (HC) group comprised healthy volunteers from the same geographic region, who were sex and age matched to the patient group.

Subjects were excluded if they had a history of psychiatric or neurological conditions, diabetes, stroke, tumors, head trauma, alcohol or substance misuse, and cardiovascular diseases. These criteria applied to all participants (NPSLE patients, non-NPSLE patients, and HCs).

### Data collection

Recorded clinical data included age, sex, complement C_3_ and C_4_ (Weinstein et al., 2021; Ayano and Horiuchi, 2023), disease duration, and SLE Disease Activity Index (SLEDAI) (Bombardier et al., 1992).

### Sample size

An a priori power analysis was conducted using G*Power 3.1.9.7 software (University of Düsseldorf, North Rhine-Westphalia, Germany) (Faul et al., 2007). According to Cohen’s criteria for effect size (Cohen, 1988), the effect size was set at *f* = 0.4, with a significance level of *α* = 0.05 and statistical power (1 − β) = 0.80, involving three groups: the NPSLE, non-NPSLE, and HC groups. Considering a 20% data attrition rate (caused by imaging artifacts and clinical dropouts), the final recruitment target was set at 83 participants, with at least 28 participants per group.

### Image acquisition

MRI scans were performed using a 3.0 T VIDA scanner (Siemens Healthineers AG, Erlangen, Bavaria, Germany) equipped with a 64-channel phased-array head coil. Participants were instructed to remain still with their heads immobilized during the scan to minimize motion. Diffusion MRI scanning was conducted in the axial plane, following a half q-space Cartesian grid sampling method with 128 diffusion directions and ten *b*-values (ranging from 0–3000 s/mm^2^). The detailed parameters included: repetition time/echo time = 6000/93 ms, field of view = 220 mm × 220 mm, slice thickness = 2.0 mm, voxel size = 2.0 × 2.0 × 2.0 mm^3^, 80 axial slices, *b*-values = 0–3000 s/mm^2^, phase encoding direction: P>>A (i.e., posterior-to-anterior direction), and total scan time = 13 minutes 35 secongs.

### Preprocessing and parameter calculations

Data preprocessing, which included head motion correction (eddy corrected) and skull stripping to minimize non-brain signal interference, was performed using NeuroDiLab software, which was developed in-house based on DIPY (Diffusion Imaging in Python, http://nipy.org/dipy). The parameters for DTI, DKI, NODDI, and MAP were also calculated using this software. Tract-based spatial statistics analysis (TBSS) analysis was conducted using FSL (FMRIB Software Library, https://fsl.fmrib.ox.ac.uk/fsl/fslwiki) (Smith et al., 2004), which involved registering fractional anisotropy (FA) data to MNI152 space and projecting these images onto the whole-brain FA skeleton. Non-FA parameters were projected onto the skeleton for voxel-wise statistical analysis.

Non-parametric permutation tests (5000 permutations) were executed using the “randomize” function in the FSL toolbox. Family-wise error correction for multiple comparisons was performed using threshold-free cluster enhancement (Smith and Nichols, 2009; Winkler et al., 2014), and clusters larger than 50 voxels were retained. XTRACT Human Connectome Project (HCP) Probabilistic Tract Atlases (Warrington et al., 2020) were used to describe the regions of each significant cluster. For cluster analysis, all metrics were averaged within each significant cluster. ROC curve analysis, including the area under the curve (AUC), was conducted to evaluate the discriminative potential of each DTI, DKI, NODDI, and MAP-MRI parameter across non-NPSLE, NPSLE, and HCs. An AUC > 0.7 was considered significant.

### Region of interest analysis

Forty-two white matter tract templates from XTRACT HCP Probabilistic Tract Atlases served as the regions of interest (ROIs) for extracting mean parameter values (DTI, DKI, NODDI, and MAP-MRI) within the corresponding regions.

### Outcomes

The results of the TBSS analysis were visually presented to illustrate regional differences. ROC curve analysis, with the AUC as the primary metric, was used to evaluate the diagnostic efficacy of significantly altered diffusion parameters—both those identified through TBSS and those highlighted in the ROI analyses. Furthermore, correlation analyses were conducted between the mean values of these significantly altered parameters within specific white matter tracts and clinical indicators, including the SLEDAI-2K score, C_3_ and C_4_ levels, and disease duration.

### Statistical analysis

Clinical data processing and analysis were performed using R software version 4.4.0 (https://www.R-project.org) (Team, 2024). Measurement data conforming to a normal distribution are presented as the mean ± standard deviation, whereas non-normally distributed measurement data are described using the median and interquartile range. Count data are expressed as numbers and percentages (*n*, %). The distribution characteristics of clinical data were assessed using the Shapiro-Wilk normality testing. Group comparisons used analysis of variance for normally distributed data or Welch’s *t*-test for non-normally distributed data, with *post hoc* Tukey–Kramer testing for multi-group comparisons. Chi-squared tests were used to evaluate sex differences. Comparisons of C_3_, C_4_, disease duration, and SLEDAI scores between non-NPSLE patients and NPSLE patients were conducted using two-sample *t*-tests. For ROI analyses across the three groups, Bonferroni correction was applied to adjust for multiple comparisons (adjusted *α* = 0.0167), and effect sizes (Cohen’s *d*) were calculated for significant differences. ROC curves and AUC values were used to assess the discriminatory ability of DTI, DKI, NODDI, and MAP-MRI metrics in each white matter tract among non-NPSLE patients, NPSLE patients, and HCs. ROC curve analysis was performed based on a support vector machine model with the following workflow: 1) stratified random sampling using the caTools package to split the dataset into training and test sets (7:3 ratio); 2) support vector machine model construction via the e1071 package, incorporating class weights to address data imbalance; and 3) model performance evaluation through ROC curve analysis and calculation of the AUC. Spearman correlation analysis was used to evaluate the relationships between mean white matter tract values (significant from the ROI analysis) and C_3_, C_4_, disease duration, or SLEDAI scores, in which *P* < 0.05 (two-tailed) was considered significant. An AUC > 0.7 was considered meaningful.

## Results

### Participant demographics

**Figure 2:**
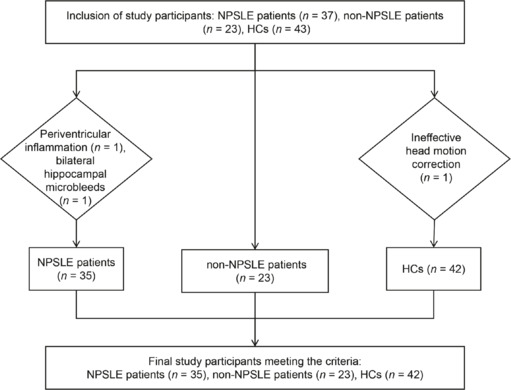
Participant selection flowchart. HCs: Healthy controls; NPSLE: neuropsychiatric systemic lupus erythematosus.

Initially, 35 participants with NPSLE, 23 participants with non-NPSLE (these numbers were limited by clinical recruitment challenges), and 42 HCs were enrolled, resulting in a total sample size of 100 participants, which exceeded the calculated requirement. After screening, 35 patients with NPSLE (mean age: 34.5 ± 12.2 years), 23 patients with non-NPSLE (mean age: 32.8 ± 10.4 years), and 42 age- and sex-matched HCs (mean age: 33.6 ± 8.9 years) met the inclusion criteria. In the NPSLE group, two subjects were excluded because of periventricular inflammation (*n* = 1) and bilateral hippocampal microbleeds (*n* = 1). Additionally, one subject from the HC group was excluded because of ineffective head motion correction. The study flowchart is shown in **Figure 2**, and specific clinical details for the participants are provided in **Table 1**.

**Table 1:** Participant demographics

Item	NPSLE group (*n* = 35)	non-NPSLE group (*n* = 23)	HC group (*n* = 42)	*Chi^2^/F/t*	*P*-value
**Clinical characteristics**					
Gender, male/female, *n*	2/33	1/22	6/36	2.502	0.286
Age, mean ± SD, yr	34.5 ± 12.2	32.8 ± 10.4	33.6 ± 8.9	0.173	0.842
Disease duration, median (IQR), mon	17 (3, 114)	3 (1.5, 26)	–	2.801	0.007*
SLEDAI-2K, mean ± SD	17.9 ± 6.7	9.4 ± 4.8	–	5.605	0.001*
Smoking, *n* (%)	4 (11.4)	2 (8.7)	2 (4.8)	1.173	0.556
C_3_, mean ± SD, g/L	0.72 ± 0.3	0.57 ± 0.22	–	2.136	0.037
C_4_, mean ± SD, g/L	0.098 ± 0.08	0.067 ± 0.054	–	1.718	0.091
**Neuropsychiatric manifestations, *n* (%)**					
Cognitive dysfunction	16 (45.7)	–	–	–	–
Cerebrovascular disease	3 (8.6)	–	–	–	–
Headache, migraine, and intracranial hypertension	5 (14.3)	–	–	–	–
Cranial neuropathy	1 (2.9)	–	–	–	–
Seizures	2 (5.7)	–	–	–	–
Mood disorders	5 (14.3)	–	–	–	–
Movement disorders	2 (0.06)	–	–	–	–
Psychosis	2 (5.7)	–	–	–	–
Acute confusional state	1 (2.9)	–	–	–	–

Key findings include significantly higher disease activity (SLEDAI) and longer disease duration in patients with NPSLE (*P < 0.05). Group comparisons were conducted using a Chi-squared test for gender, one-way analysis of variance with *post hoc* pairwise comparisons via the Tukey-Kramer test for age, and Welch’s *t*-test for SLEDAI and disease duration. “–” indicates not detected. HC: Healthy control; IQR: interquartile range; non-NPSLE: systemic lupus erythematosus without neuropsychiatric symptoms; NPSLE: neuropsychiatric systemic lupus erythematosus; SD: standard deviation; SLEDAI: systemic lupus erythematosus disease activity index.

### Tract-based spatial statistics analysis

**Figure 3:**
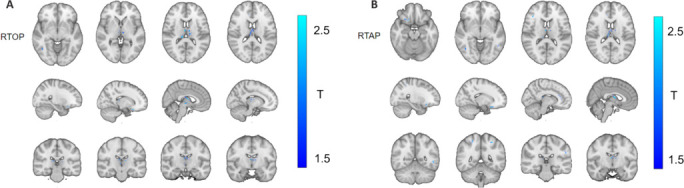
Detection images of tract-based spatial statistics. Tract-based spatial statistics results show regions of significant white matter microstructural differences between patients with neuropsychiatric systemic lupus erythematosus (NPSLE) (*n* = 35) and patients with non-NPSLE (*n* = 23), specifically within the MAP-MRI model. Blue regions indicate voxels where the NPSLE group exhibited significantly lower values compared with the non-NPSLE group for the respective MAP-MRI metrics. (A) Significant clusters for RTOP. (B) Significant clusters for RTAP. MAP-MRI: Mean apparent propagator magnetic resonance imaging; NPSLE: neuropsychiatric systemic lupus erythematosus; RTAP: return to axis probability; RTOP: return-to-origin probability.

**Figure 4:**
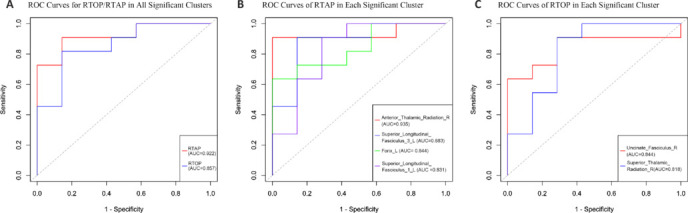
ROC curves evaluating the discriminative ability of diffusion MRI metrics to differentiate between patients with NPSLE and patients with non-NPSLE. (A) ROC curves for the averaged MAP-MRI parameters (RTOP, RTAP) within the significant clusters identified by TBSS analysis (Figure 3). (B) ROC curves for the averaged RTAP values within the significant white matter tracts identified by ROI analysis using the XTRACT human connectome project (HCP) probabilistic tract atlases. (C) ROC curves for the averaged RTOP values within the significant white matter tracts identified by the same ROI analysis. Statistical significance was set at *P* < 0.05, corrected for family-wise error using threshold-free cluster enhancement. AUC: Area under the ROC curve; MRI: magnetic resonance imaging; NPSLE: neuropsychiatric systemic lupus erythematosus; ROC: Receiver operating characteristic; ROI: region of interest; TBSS: tract-based spatial statistics.

**Figure 5:**
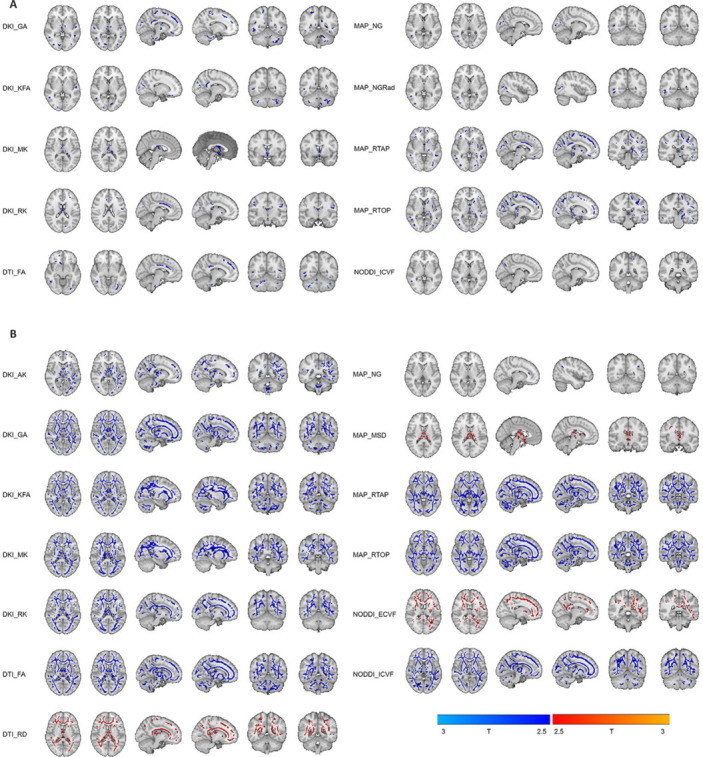
TBSS results showing widespread white matter microstructural alterations in patients with SLE compared with HCs. (A) Non-NPSLE (*n* = 23) *vs.* HCs (*n* = 42): Significant clusters showing differences in multiple diffusion metrics derived from DKI (GA, KFA, MK, RK), DTI (FA), MAP-MRI (NG, NGRad, RTOP, RTAP), and NODDI (ICVF). Blue regions indicate voxels where the non-NPSLE group showed significantly lower values compared with HCs. Key areas of abnormality include the frontal lobe, corpus callosum, temporal lobes, and cerebellum (Additional Table 1). (B) NPSLE (*n* = 35) *vs.* HCs (*n* = 42): Significant clusters showing differences in multiple diffusion metrics derived from DKI (AK, GA, KFA, MK, RK), DTI (FA, RD), MAP-MRI (NG, MSD, RTOP, RTAP), and NODDI (ECVF, ICVF). Blue regions indicate voxels where the NPSLE group showed significantly lower values compared with HCs. Red regions indicate voxels where the NPSLE group showed significantly higher values (e.g., RD, ECVF, MSD). Alterations were more extensive and diffuse compared with non-NPSLE *vs.* HCs, involving increased diffusivity and extracellular space metrics alongside decreased anisotropy and kurtosis metrics (Additional Table 1). *The patient cohort predominantly consisted of young females (accounting for over 94%). HCs were matched for age and sex. Additional details are provided in Table 1. Statistical significance was set at *P* < 0.05, corrected for family-wise error using threshold-free cluster enhancement, and further Bonferroni-corrected for the three group comparisons (*P* < 0.0167). AK: Axial kurtosis; DKI: diffusion kurtosis imaging; DTI: diffusion-tensor imaging; ECVF: extra-cellular volume fraction; FA: fractional anisotropy; GA: geodesic anisotropy; HC: healthy controls; ICVF: Intra-cellular volume fraction; KFA: kurtosis fractional anisotropy; MAP-MRI: mean apparent propagator magnetic resonance imaging; MK: mean kurtosis; MSD: mean squared displacement; NG: non-gaussianity; NGRad: non-gaussianity radial; NODDI: neurite orientation dispersion and density imaging; NPSLE: neuropsychiatric systemic lupus erythematosus; RD: radial diffusivity; RK: radial kurtosis; RTAP: return to axis probability; RTOP: return-to-origin probability; SLE: systemic lupus erythematosus; TBSS: tract-based spatial statistics analysis.

In the TBSS analysis, differences between the non-NPSLE and NPSLE groups were identified in just two parameters: return to origin probability (RTOP; *P* = 0.036, cluster size = 1416) and return to axis probability (RTAP; *P* = 0.036, cluster size = 1116) within the MAP-MRI diffusion model (**Figure 3** and **Table 2**). Notably, overlapping reductions were observed in many regions. ROC curve analysis of the varied regions (**Figure 4A**) indicated the highest AUC for RTAP (AUC = 0.922), with an optimal cutoff value of 0.39, sensitivity of 90.9%, and specificity of 85.7%. No differences were observed in other diffusion models, including DTI, DKI, and NODDI. After Bonferroni correction, multiple parameters exhibited significant differences in the non-NPSLE and NPSLE groups compared with the HC group (**Figure 5** and **Additional Table 1**)

**Table 2:** Differences in white matter tracts between non-NPSLE and NPSLE patients using TBSS based on MAP-MRI

Metrics	Cluster index	Number of voxels	Signal peaks (*x, y, z*)	Minimal *P*-value	White matter tracts
RTOP					
	1	52	9, 39, 5	0.028	Cingulum subsection: Peri-genual R; Cingulum subsection: Dorsal R
	2	55	28, 20, –20	0.012	Uncinate fasciculus R
	3	64	17, 17, –22	0.017	Uncinate fasciculus R
	4	125	3, –22, –5	0.014	Superior thalamic radiation R
					Anterior thalamic radiation R
	5	935	–5, 1, 7	0.006	Superior thalamic radiation R
					Superior thalamic radiation L
					Fornix R
					Fornix L
					Anterior thalamic radiation R
					Anterior thalamic radiation L
RTAP					
	1	50	46, 28, 8	0.034	Superior longitudinal fasciculus 3 R
	2	59	–13, –7, 17	0.012	Anterior thalamic radiation L
					Superior thalamic radiation L
	3	61	–56, –29, 27	0.025	Arcuate fasciculus L
					Superior longitudinal fasciculus 3 L
	4	63	–48, –55, –6	0.012	Arcuate fasciculus L
					Inferior longitudinal fasciculus L
	5	69	31, –43, 46	0.017	Superior longitudinal fasciculus 1 R
	6	83	–36, –41, 47	0.01	Superior longitudinal fasciculus 1 L
	7	85	17, 16, –22	0.012	Uncinate fasciculus R
	8	94	10, 38, 4	0.021	Cingulum subsection: Dorsal R
					Cingulum subsection: Peri-genual R
	9	99	28, 20, –20	0.011	Uncinate fasciculus R
	10	378	–4, –2, 11	0.004	Anterior thalamic radiation L
					Anterior thalamic radiation R
					Fornix L
					Fornix R

L: Left side; MAP-MRI: mean apparent propagator magnetic resonance imaging; NPSLE: neuropsychiatric systemic lupus erythematosus; R: right side; RTAP: return to axis probability; RTOP: return to origin probability; TBSS: tract-based spatial statistics analysis.

### Atlas-based regions of interest analysis

**Figure 6:**
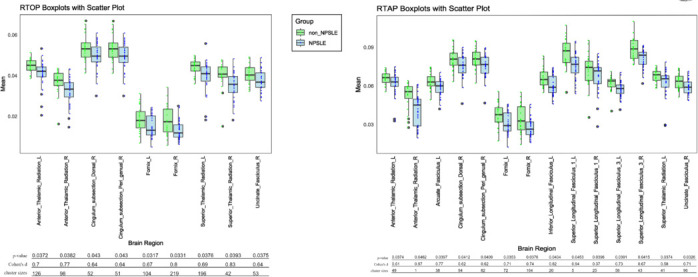
Mean RTOP and RTAP values of significant XTRACT tracts were analyzed using Student’s *t*-test with threshold-free cluster enhancement correction for family-wise error. Scatterplots illustrate the mean RTOP and RTAP values of various XTRACT tracts for each participant. Boxplots display the median, mean, minimum, maximum, and interquartile range. L: Left; R: right; RTAP: return to axis probability; RTOP: return-to-origin probability.

Two key metrics obtained from TBSS analysis (RTOP and RTAP) were studied through atlas-based ROI analysis. After family-wise error correction, both metrics exhibited significant differences between the NPSLE and non-NPSLE groups (**Figure 6**). In the RTOP analysis, compared with the non-NPSLE group, the NPSLE group exhibited reductions in the bilateral superior thalamic radiation (STR), bilateral anterior thalamic radiation (ATR), bilateral fornix, right uncinate fasciculus (UF), and right cingulum. In the RTAP analysis, the NPSLE group demonstrated decreases in the bilateral ATR, left STR, right cingulum, right UF, bilateral fornix, bilateral superior longitudinal fasciculus (SLF), left inferior longitudinal fasciculus, and left arcuate fasciculus. ROC curve analysis was performed on these regions (**Figure 4B** and **C**). The RTOP value in the right UF showed the largest AUC (AUC = 0.844) for distinguishing NPSLE from non-NPSLE patients, with an optimal cutoff of −0.26 (sensitivity = 63.6%, specificity = 100%). Although the right ATR exhibited the highest AUC value, its cluster size was 1 and it was therefore excluded. The RTAP metric in the left SLF-3 demonstrated the highest discriminative capability (AUC = 0.883), with an optimal cutoff of 0.31 (sensitivity = 90.9%, specificity = 85.7%). Bonferroni correction for the XTRACT HCP Probabilistic Tract Atlases revealed that detailed AUC values for the white matter regions differed in the non-NPSLE and NPSLE groups compared with the HC group (**Additional Table 2**).

### Correlation analysis

**Figure 7:**
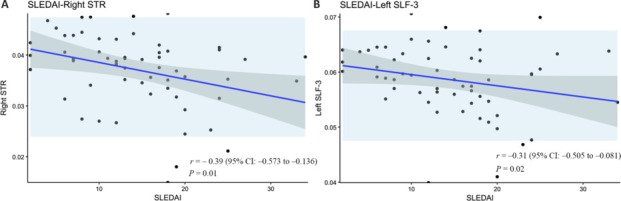
Results of the correlation analysis. (A) Spearman correlation analysis of SLEDAI scores and right STR of RTOP in the NPSLE group. (B) Spearman correlation analysis of SLEDAI scores and left SLF-3 of RTOP in the NPSLE group. NPSLE: Neuropsychiatric systemic lupus erythematosus; RTOP: return-to-origin probability; SLEDAI: systemic lupus erythematosus disease activity index; SLF-3: superior longitudinal fasciculus-3; STR: superior thalamic radiation.

As shown in **Figure 7**, both the RTOP in the right STR and the RTAP in the left SLF-3 exhibited negative correlations with the SLEDAI score. No significant correlations were observed between the RTOP/RTAP values and the variables of C3, C4, and disease duration.

## Discussion

The present study addressed two critical issues in the neuroimaging of SLE: first, the comparative value of multiple advanced diffusion models (DTI, DKI, NODDI, and MAP-MRI) for detecting white matter microstructural alterations; and second, the specific injury patterns distinguishing NPSLE from non-NPSLE patients. Unlike previous studies that primarily relied on DTI or excluded NPSLE cohorts, our work provides a comprehensive multi-model characterization of white matter damage in SLE patients, identifying MAP-MRI as the most sensitive technique for distinguishing NPSLE-specific pathology. Furthermore, we have established a clear link between microstructural integrity and clinical disease activity, and propose the RTAP parameter in the left SLF-3 as a novel, clinically actionable imaging biomarker.

Our findings have important implications for neuroregeneration research. The observed reductions in RTOP and RTAP suggest damage to axonal integrity and myelin organization in patients with NPSLE. Alterations in specific white matter tracts were identified through ROI analysis, providing potential anatomical targets for future studies into neural injury in NPSLE. The strong correlation between diffusion metrics and SLEDAI scores further indicates that controlling disease activity in patients with NPSLE may help to slow the progression of neural damage. The parameters defined in the current study may therefore serve as objective biomarkers for monitoring neural injury or repair in NPSLE.

As a systemic disease, SLE can damage the nervous system and cause NPSLE. Studies have found that patients with NPSLE have a higher probability of exhibiting WMH in the corpus callosum, the right superior longitudinal fasciculus, and the corona radiata compared to non-NPSLE patients. Additionally, patients with NPSLE show larger periventricular WMH volumes and more complex WMH shapes, a finding that is more pronounced in patients with NPSLE with autoimmune pathways. However, not all patients with SLE had WMH.

In the present study, we analyzed white matter microstructure in SLE using multiple diffusion models and identified significant changes in the white matter microstructure of patients with SLE (with or without neuropsychiatric involvement). Additionally, the MAP diffusion model was able to distinguish white matter microstructural differences between the non-NPSLE and NPSLE groups, as evidenced by lower RTOP and RTAP values in patients with NPSLE.

Previous studies have primarily used the DTI diffusion model to investigate white matter microstructure changes in patients with SLE. The main findings indicate lower FA values in the fornix radiation, inferior longitudinal fasciculus, superior longitudinal fasciculus, and corpus callosum (Correa et al., 2016; Shastri et al., 2016; Costallat et al., 2018; Zhou et al., 2021; Wang et al., 2023a; Bai et al., 2024). In the present study, we also observed differences in these regions. Moreover, the application of multiple diffusion models allowed for the interpretation and analysis of these differences from various perspectives. A decrease in FA values suggests white matter fiber loss. Additionally, the decline in average and radial kurtosis, also referred to as NG/NGRad, further supports the evidence of white matter fiber damage under the non-Gaussian diffusion of water molecules. This is primarily indicated by a reduction in the degree of diffusion restriction perpendicular to the axonal direction. The decrease in intracellular volume fraction further confirms the reduction in neurite density. In addition, the decrease in RTOP and RTAP indicates a reduction in the degree of diffusion restriction along the axonal direction. However, the areas of difference for each parameter are not consistent, reflecting the advantages and disadvantages of various diffusion models and parameters; these should be used in conjunction to compensate for their different limitations. Although Hu et al. (2024b) used three diffusion models—DTI, DKI, and NODDI, they did not include patients with NPSLE, examining the white matter microstructure of patients with non-NPSLE only.

Notably, in NPSLE patients, all diffusion parameters except for NG/NGRad exhibited more extensive areas of difference. This suggests that white matter damage in NPSLE patients is diffuse and multi-dimensional. It is also important to highlight that the NG parameters reflecting the non-Gaussian diffusion of water molecules showed almost no changes. This implies that SLE causes widespread damage to white matter fibers, thereby rendering the diffusion of water molecules more Gaussian.

In the present study, only the RTOP and RTAP parameters differed between NPSLE and non-NPSLE patients. Compared with non-NPSLE patients, NPSLE patients exhibited significantly lower RTOP and RTAP values. This finding indicates that the degree of diffusion restriction along the axonal direction is smaller in NPSLE patients; this suggests more severe white matter fiber damage, which can impair signal conduction.

We further used the XTRACT HCP Probabilistic Tractography Atlas to perform ROI segmentation on white matter regions with significant differences. We analyzed the clinical significance of the decreased RTOP and RTAP values in these regions by considering the specific functions of the involved fiber tracts in the nervous system and the neuropsychiatric symptoms of NPSLE patients. Additionally, we assessed the diagnostic efficacy of each region using ROC curve analysis. We demonstrated that the RTAP of the left SLF-3 had the best discriminative ability, suggesting that SLF-3 fibers may serve as potential biomarkers for NPSLE.

The STR is a key fiber tract that connects the thalamus and the cerebral cortex. It primarily transmits sensory information and participates in complex cognitive tasks (An et al., 2023). The SLF is a dorsal fiber bundle that connects multiple lobes of the brain, coordinating cognitive, language, and attention functions. Its SLF-3 subregion is associated with cognitive function in patients with cerebral small vessel disease (Szeszko et al., 2018; Mamah et al., 2024; Wang et al., 2024). The ATR mediates memory, executive function, and depressive symptoms (Gutman et al., 2009; Ribeiro et al., 2024). The fornix, which connects the hippocampus and hypothalamus, supports memory consolidation and emotional regulation (Liu et al., 2020). The cingulum shows microstructural alterations in depression (Hu et al., 2024a). The UF integrates emotion and verbal memory (Zheng et al., 2018; Xu et al., 2023), whereas the inferior longitudinal fasciculus processes visual memory (Herbet et al., 2018; Nostadt et al., 2024). The arcuate fasciculus supports language function through the Broca–Wernicke connection pathway and connects different brain regions to maintain attention (Becker et al., 2022). In summary, the fiber tract regions with reduced RTOP and RTAP values in NPSLE patients in the current study may help to explain their neuropsychiatric manifestations. Nonetheless, the importance of the altered fiber tract regions in the nervous system of the non-NPSLE and NPSLE groups compared with the HC group is not discussed here.

In the correlation analysis, the RTOP value of the right STR and the RTAP value of the left SLF-3 were both negatively correlated with the SLEDAI score. This indicates that white matter fiber damage in SLE patients is associated with SLE disease activity. Even in the absence of neuropsychiatric symptoms, substantial white matter microstructure changes may already be present in SLE patients with high SLEDAI scores.

As a novel imaging biomarker, the RTAP of SLF-3 can be effectively integrated into the current NPSLE diagnostic process to address the limitations of SLEDAI. For SLE patients with an SLEDAI score ≥ 6 and neuropsychiatric symptoms, MAP-MRI detection can provide objective evidence to support a diagnosis of NPSLE. In disease stratification, RTAP can complement SLEDAI. A low SLEDAI with marked RTAP reduction indicates subclinical neuroinflammation that requires enhanced central nervous system-targeted therapy. Conversely, a high SLEDAI with severe RTAP decline reflects concurrent systemic inflammation and neuroinflammation.

## Limitations

The present study has some limitations. First, the relatively small cohort size (NPSLE: *n* = 35; non-NPSLE: *n* = 23) restricted our ability to conduct subgroup analyses. For example, we were unable to distinguish between various manifestations of NPSLE, such as cognitive impairment, cerebrovascular disease, and epilepsy. Second, medications for SLE (used in both NPSLE and non-NPSLE patients) may affect diffusion metrics. Third, although patients with a history of stroke were excluded, the common occurrence of microthrombosis in SLE may lead to chronic hypoperfusion, potentially confounding the results. Fourth, although Bonferroni correction controls for Type I errors, it also increases the risk of Type II errors, possibly resulting in the missed detection of other microstructural white matter injuries and multiparametric interaction effects. Finally, the lack of long-term follow-up data hinders our understanding of the temporal evolution of white matter injuries.

## Conclusion

Significant differences were observed between the non-NPSLE and NPSLE groups in the MAP diffusion model, particularly concerning the RTAP parameter. ROI analysis revealed that the RTAP value of the left SLF-3 region exhibited the strongest classification ability for distinguishing between the two groups. Additionally, the RTOP value of the right STR and the RTAP value of the left SLF-3 were both negatively correlated with each other and positively correlated with the SLEDAI score. This indicates that the degree of white matter injury is closely related to disease severity. Together, these findings suggest that MAP-MRI metrics—especially the RTAP parameter—have the potential to serve as biomarkers for white matter microstructural changes in NPSLE patients. By identifying the most characteristic fiber tracts in the left SLF-3 region, these metrics provide important evidence for clinical diagnosis.

## Additional files:

***Additional Table 1:*** *TBSS analysis of white matter fiber tracts between HC and non-NPSLE.*

Additional Table 1 A TBSS Analysis of White Matter Fiber Tracts Between HC and Non-NPSLE

***Additional Tabel 2:*** *The AUC of white matter fiber tract metrics between HC and non-NPSLE.*

Additional Table 2A The AUC of White Matter Fiber Tract Metrics Between HC and Non-NPSLE

## Data Availability

*All relevant data are within the paper and its Additional files*.
