# Supplementary material for: Detection of white matter microstructural changes in patients with systemic lupus erythematosus based on multiple diffusion models and related diffusion metrics
Source: Neural Regen Res. 2025 Nov 25;21(6):2467–74. doi: 10.4103/NRR.NRR-D-25-00730 (PMC13211794; doi:10.4103/NRR.NRR-D-25-00730)
Supplement: Supplementary file 1 [file NRR-21-2467_Suppl1.pdf]

Additional Table 1 A TBSS Analysis of White Matter Fiber Tracts Between HC and Non-NPSLE

Metrics	Cluster index	Number of voxels	Signal peaks (x,y,z)	Maximally 1-p value	White matter tracts
FA					
	1	53	10 -83 29	0.992	Forceps Major
	2	63	-26 23 -15	0.99	Uncinate Fasciculus L
	3	71	40 19 36	0.995	Superior Longitudinal Fasciculus 2 R
	4	71	-27 -19 63	0.995	Corticospinal Tract L
					Superior Thalamic Radiation L
	5	73	14 52 -11	0.998	Anterior Thalamic Radiation R
					Forceps Minor
					Inferior Fronto-Occipital Fasciculus R
					Uncinate Fasciculus R
	6	74	-7 35 13	0.999	Cingulum subsection: Dorsal L
					Cingulum subsection: Peri-genual L
	7	77	-8 -3 37	0.997	Cingulum subsection: Dorsal L
	8	82	-11 -85 34	0.997	Forceps Major
					Middle Longitudinal Fasciculus L
					Optic Radiation L
					Vertical Occipital Fasciculus L
	9	89	31 22 -18	0.995	Uncinate Fasciculus R
	10	94	-31 2 54	0.993	Superior Longitudinal Fasciculus 2 L
	11	96	-33 -51 -50	0.995	Middle Cerebellar Peduncle
	12	100	49 -56 -8	0.996	Inferior Longitudinal Fasciculus R
	13	108	8 -80 2	0.997	Forceps Major
					Inferior Fronto-Occipital Fasciculus R
					Optic Radiation R
	14	108	34 -41 46	0.999	Superior Longitudinal Fasciculus 1 R

15	122	-48	15	13	0.999	Arcuate Fasciculus L
						Frontal Aslant Tract L
						Superior Longitudinal Fasciculus 3 L
16	127	-60	-30	10	0.996	Arcuate Fasciculus L
						Acoustic Radiation L
						Middle Longitudinal Fasciculus L
17	144	-11	-34	-40	0.997	Corticospinal Tract L
						Middle Cerebellar Peduncle
18	149	-11	-38	29	0.999	Cingulum subsection: Dorsal L
19	164	-35	-64	-34	0.99	Middle Cerebellar Peduncle
20	211	24	-93	10	0.999	Forceps Major
						Middle Longitudinal Fasciculus R
						Optic Radiation R
						Vertical Occipital Fasciculus R
21	216	16	9	-17	0.997	Cingulum subsection: Dorsal R
						Cingulum subsection: Peri-genual R
						Uncinate Fasciculus R
22	246	37	-51	-46	0.999	Middle Cerebellar Peduncle
23	305	-40	-72	-5	0.999	Forceps Major
						Inferior Fronto-Occipital Fasciculus L
						Inferior Longitudinal Fasciculus L
						Vertical Occipital Fasciculus L
24	348	22	-58	-28	0.998	Middle Cerebellar Peduncle
25	475	-11	31	45	0.999	Anterior Thalamic Radiation L
						Cingulum subsection: Dorsal L
						Frontal Aslant Tract L
						Forceps Minor
						Inferior Fronto-Occipital Fasciculus L
						Superior Longitudinal Fasciculus 1 LSu

						perior Thalamic Radiation L
RK						
1	50	-11	9	-2	0.995	Anterior Thalamic Radiation L
2	59	-27	41	14	0.991	Forceps Minor
						Superior Longitudinal Fasciculus 2 L
3	61	56	-26	-19	0.996	Inferior Longitudinal Fasciculus R
4	64	-54	-28	28	0.995	Arcuate Fasciculus L
						Superior Longitudinal Fasciculus 3 L
5	72	13	60	1	0.994	Anterior Thalamic Radiation R
						Forceps Minor
						Inferior Fronto-Occipital Fasciculus R
						Uncinate Fasciculus R
6	73	-10	-34	35	0.992	Cingulum subsection: Dorsal L
7	82	-17	-72	14	0.996	Forceps Major
8	83	52	-29	42	0.995	Arcuate Fasciculus R
						Superior Longitudinal Fasciculus 3 R
9	90	38	20	37	0.996	Superior Longitudinal Fasciculus 2 R
10	92	11	30	18	0.997	Anterior Thalamic Radiation R
						Cingulum subsection: Dorsal R
						Cingulum subsection: Peri-genual R
11	95	-39	30	26	0.996	Superior Longitudinal Fasciculus 2 L
12	105	31	20	29	0.995	Superior Longitudinal Fasciculus 2 R
13	107	48	-15	34	0.993	Arcuate Fasciculus R
						Superior Longitudinal Fasciculus 3 R
14	112	50	1	37	0.996	Superior Longitudinal Fasciculus 2 R
15	154	-21	-33	3	0.996	Acoustic Radiation L
						Fornix L
						Optic Radiation L
16	197	-7	30	18	0.999	Cingulum subsection: Dorsal L

							Cingulum subsection: Peri-genual L
	17	241	-56	-11	22	0.996	Arcuate Fasciculus L
							Superior Longitudinal Fasciculus 2 L
							Superior Longitudinal Fasciculus 3 L
	18	348	1	-14	19	0.998	Anterior Commissure
							Fornix L
							Fornix R
KFA							
	1	64	-25	-55	-36	0.995	Middle Cerebellar Peduncle
	2	75	31	-73	-7	0.997	Vertical Occipital Fasciculus R
	3	78	-8	-2	66	0.994	Superior Longitudinal Fasciculus 1 L
							Superior Thalamic Radiation L
	4	80	-35	-60	-36	0.997	Middle Cerebellar Peduncle
	5	88	-10	-81	2	0.996	Forceps Major
							Inferior Fronto-Occipital Fasciculus L
							Optic Radiation L
	6	94	-17	23	49	0.995	Frontal Aslant Tract L
							Superior Longitudinal Fasciculus 1 L
							Superior Thalamic Radiation L
	7	97	41	21	33	0.998	Superior Longitudinal Fasciculus 2 R
	8	99	-25	-63	49	1	Superior Longitudinal Fasciculus 1 L
							Superior Longitudinal Fasciculus 2 L
	9	100	-20	-54	-39	0.995	Middle Cerebellar Peduncle
	10	118	37	-51	-46	1	Middle Cerebellar Peduncle
	11	133	16	-95	14	1	Forceps Major
							Inferior Fronto-Occipital Fasciculus R
							Middle Longitudinal Fasciculus R
							Optic Radiation R
							Vertical Occipital Fasciculus R

	12	134	-55	-10	1	0.997	Acoustic Radiation L
							Middle Longitudinal Fasciculus L
	13	140	-42	-25	52	0.997	Superior Longitudinal Fasciculus 2 L
	14	154	13	-47	9	1	Cingulum subsection: Dorsal R
							Forceps Major
	15	169	16	36	-21	1	Uncinate Fasciculus R
	16	242	-30	-59	-51	1	Middle Cerebellar Peduncle
	17	248	20	-69	13	0.998	Forceps Major
							Middle Longitudinal Fasciculus R
							Optic Radiation R
							Vertical Occipital Fasciculus R
	18	284	-47	-6	-25	0.998	Inferior Longitudinal Fasciculus L
	19	360	-7	35	13	1	Cingulum subsection: Dorsal L
							Cingulum subsection: Peri-genual L
MK							
	1	221	2	-2	-11	0.995	Anterior Commissure
							Cingulum subsection: Dorsal R
							Fornix L
							Fornix R
	2	417	1	-14	19	0.998	Anterior Thalamic Radiation L
							Anterior Thalamic Radiation R
							Fornix L
							Fornix R
							Superior Thalamic Radiation L
							Superior Thalamic Radiation R
GA							
	1	50	-50	-40	-14	0.997	Arcuate Fasciculus L
							Inferior Longitudinal Fasciculus L
	2	50	9	35	15	0.999	Cingulum subsection: Dorsal R

						Cingulum subsection: Peri-genual R
3	51	13	-47	9	0.999	Forceps Major
						Cingulum subsection: Dorsal R
4	53	55	-7	-24	0.996	Inferior Longitudinal Fasciculus R
5	56	-9	-13	-2	0.996	Superior Thalamic Radiation L
						Anterior Thalamic Radiation L
6	56	34	-84	-1	0.998	Vertical Occipital Fasciculus R
7	60	-14	-50	24	0.999	Cingulum subsection: Dorsal L
8	62	-32	-85	1	0.999	Forceps Major
						Vertical Occipital Fasciculus L
9	63	-47	4	-17	0.997	Middle Longitudinal Fasciculus L
						Uncinate Fasciculus L
10	63	6	-7	-11	0.996	Anterior Thalamic Radiation R
11	65	47	13	6	0.999	Arcuate Fasciculus R
						Frontal Aslant Tract R
						Superior Longitudinal Fasciculus 3 R
12	65	55	6	9	0.999	Arcuate Fasciculus R
						Frontal Aslant Tract R
						Superior Longitudinal Fasciculus 3 R
13	65	60	-26	-10	0.997	Inferior Longitudinal Fasciculus R
14	66	-38	15	38	0.999	Superior Longitudinal Fasciculus 2 L
15	69	53	3	-8	0.997	Acoustic Radiation R
						Middle Longitudinal Fasciculus R
16	73	-56	-27	24	0.999	Arcuate Fasciculus L
						Superior Longitudinal Fasciculus 3 L
17	73	-23	16	-18	0.994	Uncinate Fasciculus L
18	75	40	4	-35	0.998	Inferior Longitudinal Fasciculus R
19	77	31	-62	-11	0.999	Inferior Fronto-Occipital Fasciculus R
						Vertical Occipital Fasciculus R

20	89	22	-34	63	0.996	Corticospinal Tract R
21	95	-8	36	-19	0.999	Cingulum subsection: Dorsal L
						Forceps Minor
						Uncinate Fasciculus L
22	100	-10	-3	11	0.995	Superior Thalamic Radiation L
						Anterior Thalamic Radiation L
						Fornix L
23	101	55	-26	-20	0.999	Inferior Longitudinal Fasciculus R
24	109	-30	-80	-9	0.997	Forceps Major
						Vertical Occipital Fasciculus L
25	112	-48	15	12	0.998	Arcuate Fasciculus L
						Frontal Aslant Tract L
						Superior Longitudinal Fasciculus 3 L
26	113	-44	-10	-35	0.995	Fornix L
						Inferior Fronto-Occipital Fasciculus L
						Inferior Longitudinal Fasciculus L
						Middle Longitudinal Fasciculus L
						Uncinate Fasciculus L
27	119	40	19	36	0.999	Superior Longitudinal Fasciculus 2 R
28	122	-31	10	51	0.999	Superior Longitudinal Fasciculus 2 L
29	122	-10	-89	15	0.995	Vertical Occipital Fasciculus L
						Optic Radiation L
						Middle Longitudinal Fasciculus L
						Forceps Major
30	136	31	22	-18	0.998	Uncinate Fasciculus R
						Inferior Fronto-Occipital Fasciculus R
31	136	46	-55	23	0.999	Superior Longitudinal Fasciculus 2 R
						Superior Longitudinal Fasciculus 3 R
32	143	-25	-25	-23	0.999	Cingulum subsection: Temporal L

33	151	9	-22	7	0.996	Anterior Thalamic Radiation R
						Superior Thalamic Radiation R
34	154	-56	-11	29	0.999	Superior Longitudinal Fasciculus 3 L
35	160	8	-81	1	0.999	Forceps Major
						Inferior Fronto-Occipital Fasciculus R
						Optic Radiation R
36	161	-10	-3	63	0.996	Frontal Aslant Tract L
						Superior Longitudinal Fasciculus 1 L
						Superior Thalamic Radiation L
37	169	-60	-30	10	0.998	Arcuate Fasciculus L
						Acoustic Radiation L
						Middle Longitudinal Fasciculus L
38	174	6	-70	-23	0.998	Middle Cerebellar Peduncle
39	183	-31	-48	-45	0.999	Middle Cerebellar Peduncle
40	192	-48	-17	4	0.996	Acoustic Radiation L
						Middle Longitudinal Fasciculus L
						Uncinate Fasciculus L
41	201	-40	-63	-13	0.999	Inferior Longitudinal Fasciculus L
						Vertical Occipital Fasciculus L
42	201	60	-30	12	0.999	Arcuate Fasciculus R
						Acoustic Radiation R
						Middle Longitudinal Fasciculus R
43	213	-33	-51	-50	0.999	Middle Cerebellar Peduncle
44	235	-11	-39	29	0.999	Cingulum subsection: Dorsal L
45	235	16	8	-17	0.999	Anterior Thalamic Radiation R
						Cingulum subsection: Dorsal R
						Cingulum subsection: Peri-genual R
						Inferior Fronto-Occipital Fasciculus R
						Uncinate Fasciculus R

	46	240	54	-53	-1	0.999	Inferior Longitudinal Fasciculus R
	47	258	34	-41	40	0.999	Superior Longitudinal Fasciculus 1 R
	48	269	-33	-25	49	0.999	Corticospinal Tract L
							Superior Longitudinal Fasciculus 2 L
							Superior Thalamic Radiation L
	49	355	-7	-36	63	0.999	Corticospinal Tract L
							Superior Longitudinal Fasciculus 1 L:
	50	479	-48	-1	-29	1	Arcuate Fasciculus L
							Inferior Longitudinal Fasciculus L
	51	496	-5	-33	-45	0.999	Corticospinal Tract L
							Middle Cerebellar Peduncle
	52	529	-9	55	27	0.998	Anterior Thalamic Radiation L
							Cingulum subsection: Dorsal L
							Frontal Aslant Tract L
							Forceps Minor
							Inferior Fronto-Occipital Fasciculus L
							Superior Longitudinal Fasciculus 1 L
							Superior Thalamic Radiation L
	53	712	16	-95	14	1	Forceps Major
							Middle Longitudinal Fasciculus R
							Optic Radiation R
							Superior Longitudinal Fasciculus 1 R
							Vertical Occipital Fasciculus R
NG							
	1	77	-56	-22	-17	0.998	Inferior Longitudinal Fasciculus L
	2	78	-8	-76	19	0.993	Forceps Major
	3	102	-11	-94	6	0.995	Forceps Major
							Optic Radiation L
	4	116	-40	2	-34	0.995	Inferior Longitudinal Fasciculus L

NGRad							
	1	67	-39	2	-34	0.996	Inferior Longitudinal Fasciculus L
	2	85	-56	-22	-17	0.997	Inferior Longitudinal Fasciculus L
	3	140	43	-73	9	0.995	Vertical Occipital Fasciculus R
RTOP							
	1	50	-42	33	-1	0.997	Uncinate Fasciculus L
							Inferior Fronto-Occipital Fasciculus L
							Anterior Thalamic Radiation L
							Arcuate Fasciculus L
	2	50	-52	-49	2	0.992	Arcuate Fasciculus L
							Inferior Longitudinal Fasciculus L
	3	52	-34	0	35	0.991	Superior Longitudinal Fasciculus 2 L
	4	55	-27	-40	32	0.997	Middle Longitudinal Fasciculus L
							Superior Longitudinal Fasciculus 2 L
	5	56	-11	-89	-7	0.992	Forceps Major
							Inferior Fronto-Occipital Fasciculus L
							Optic Radiation L
	6	61	-7	23	23	0.996	Cingulum subsection: Dorsal L
							Cingulum subsection: Peri-genual L
	7	62	-44	26	3	0.997	Arcuate Fasciculus L
							Superior Longitudinal Fasciculus 3 L
	8	64	-26	-71	1	0.992	Forceps Major
							Inferior Fronto-Occipital Fasciculus L
							Middle Longitudinal Fasciculus L
							Optic Radiation L
	9	64	-43	-44	7	0.99	Arcuate Fasciculus L
							Middle Longitudinal Fasciculus L
	10	65	38	30	-4	0.991	Inferior Fronto-Occipital Fasciculus R
							Superior Longitudinal Fasciculus 3 R

11	65	11	-54	59	0.996	Superior Longitudinal Fasciculus 1 R
12	67	36	25	-11	0.997	Uncinate Fasciculus R
13	68	48	14	7	0.997	Arcuate Fasciculus R
						Frontal Aslant Tract R
						Superior Longitudinal Fasciculus 3 R
14	69	32	-43	50	0.997	Superior Longitudinal Fasciculus 1 R
15	70	-19	4	-12	0.998	Cingulum subsection: Dorsal L
						Inferior Fronto-Occipital Fasciculus L
						Uncinate Fasciculus L
16	72	-29	-61	40	0.996	Superior Longitudinal Fasciculus 1 L
						Superior Longitudinal Fasciculus 2 L
17	76	-49	15	10	0.997	Arcuate Fasciculus L
						Frontal Aslant Tract L
						Superior Longitudinal Fasciculus 3 L
18	80	-8	-46	28	0.996	Cingulum subsection: Dorsal L
19	80	-26	-65	13	0.993	Forceps Major
						Middle Longitudinal Fasciculus L
						Optic Radiation L
20	81	53	-41	-18	0.991	Inferior Longitudinal Fasciculus R
21	81	-13	-84	33	0.993	Forceps Major
						Middle Longitudinal Fasciculus L
						Optic Radiation L
						Vertical Occipital Fasciculus L
22	86	-52	-50	25	0.997	Arcuate Fasciculus L
						Middle Longitudinal Fasciculus L:
						Superior Longitudinal Fasciculus 3 L
23	95	-13	58	-3	0.994	Anterior Thalamic Radiation L
						Cingulum subsection: Dorsal L
						Forceps Minor

						Inferior Fronto-Occipital Fasciculus L
						Uncinate Fasciculus L
24	104	40	21	34	0.997	Superior Longitudinal Fasciculus 2 R
25	113	-35	8	-33	0.995	Fornix L
						Uncinate Fasciculus L
26	120	-34	-9	0	0.994	Inferior Fronto-Occipital Fasciculus L
27	128	-41	-8	-37	0.997	Cingulum subsection: Temporal L
						Fornix L
						Inferior Longitudinal Fasciculus L
28	133	44	-73	10	0.999	Vertical Occipital Fasciculus R
29	134	-60	-30	10	0.993	Arcuate Fasciculus L
						Acoustic Radiation L
						Middle Longitudinal Fasciculus L
30	153	14	55	18	0.995	Anterior Thalamic Radiation R
						Cingulum subsection: Dorsal R
						Forceps Minor
						Inferior Fronto-Occipital Fasciculus R
						Uncinate Fasciculus R
31	155	-13	12	-4	0.992	Anterior Thalamic Radiation L
						Inferior Fronto-Occipital Fasciculus L
32	161	46	-55	32	0.997	Superior Longitudinal Fasciculus 2 R
						Superior Longitudinal Fasciculus 3 R
33	171	-51	-38	-18	0.996	Arcuate Fasciculus L:
						Inferior Fronto-Occipital Fasciculus L
						Inferior Longitudinal Fasciculus L
34	179	-44	6	14	0.996	Arcuate Fasciculus L
						Frontal Aslant Tract L
						Superior Longitudinal Fasciculus 3 L
35	185	-44	-59	34	0.999	Superior Longitudinal Fasciculus 2 L

						Superior Longitudinal Fasciculus 3 L
36	194	61	-26	-9	0.997	Arcuate Fasciculus R
						Inferior Longitudinal Fasciculus R
37	202	-56	-6	15	0.996	Arcuate Fasciculus L
						Superior Longitudinal Fasciculus 3 L
38	210	12	7	58	0.996	Frontal Aslant Tract R
						Superior Longitudinal Fasciculus 1 R
						Superior Thalamic Radiation R
39	259	52	-55	-1	1	Inferior Longitudinal Fasciculus R
40	286	-28	21	-19	0.996	Anterior Thalamic Radiation L
						Inferior Fronto-Occipital Fasciculus L
						Uncinate Fasciculus L
41	324	-50	-36	41	0.997	Arcuate Fasciculus L
						Superior Longitudinal Fasciculus 2 L
						Superior Longitudinal Fasciculus 3 L
42	327	-42	-25	10	0.997	Acoustic Radiation L
						Middle Longitudinal Fasciculus L
						Uncinate Fasciculus L
43	329	-36	-31	55	0.997	Corticospinal Tract L
						Superior Longitudinal Fasciculus 1 L
						Superior Longitudinal Fasciculus 2 L
						Superior Longitudinal Fasciculus 3 L
44	333	-35	-84	1	0.997	Forceps Major
						Inferior Fronto-Occipital Fasciculus L
						Middle Longitudinal Fasciculus L
						Vertical Occipital Fasciculus L
45	390	-11	-94	6	0.997	Forceps Major
						Middle Longitudinal Fasciculus L
						Optic Radiation L

						Vertical Occipital Fasciculus L
46	468	-39	3	-34	0.998	Arcuate Fasciculus L
						Fornix L
						Inferior Longitudinal Fasciculus L
						Middle Longitudinal Fasciculus L
						Uncinate Fasciculus L
47	478	18	-95	14	0.997	Forceps Major
						Middle Longitudinal Fasciculus R
						Optic Radiation R
						Vertical Occipital Fasciculus R
48	518	-36	31	21	0.997	Superior Longitudinal Fasciculus 2 L
49	596	-6	-71	34	0.997	Superior Longitudinal Fasciculus 1 L
50	786	-7	-4	67	0.997	Anterior Thalamic Radiation L
						Cingulum subsection: Dorsal L
						Corticospinal Tract L
						Frontal Aslant Tract L
						Forceps Minor
						Inferior Fronto-Occipital Fasciculus L
						Superior Longitudinal Fasciculus 1 L
						Superior Thalamic Radiation L
51	891	-38	-7	51	0.999	Corticospinal Tract L
						Superior Longitudinal Fasciculus 1 L
						Superior Longitudinal Fasciculus 2 L
						Superior Thalamic Radiation L
52	1600	4	2	3	0.999	Anterior Commissure
						Acoustic Radiation L
						Anterior Thalamic Radiation L
						Anterior Thalamic Radiation R
						Cingulum subsection: Dorsal L

							Corticospinal Tract L
							Fornix L
							Fornix R
							Optic Radiation L
							Superior Thalamic Radiation L
							Superior Thalamic Radiation R
RTAP							
	1	50	24	-93	10	0.999	Forceps Major
							Optic Radiation R
							Vertical Occipital Fasciculus R
	2	50	33	49	1	0.995	Anterior Thalamic Radiation R
							Forceps Minor
							Superior Longitudinal Fasciculus 3 R
	3	52	-32	-51	27	0.998	Middle Longitudinal Fasciculus L
							Superior Longitudinal Fasciculus 2 L
	4	53	-48	1	-15	0.992	Middle Longitudinal Fasciculus L
							Uncinate Fasciculus L
	5	55	-24	-52	5	0.997	Forceps Major
	6	55	-35	-62	23	0.992	Inferior Fronto-Occipital Fasciculus L
							Middle Longitudinal Fasciculus L
							Optic Radiation L
							Superior Longitudinal Fasciculus 1 L
	7	56	-44	26	4	0.995	Arcuate Fasciculus L
							Superior Longitudinal Fasciculus 3 L
	8	58	10	36	13	0.991	Cingulum subsection: Dorsal R
							Cingulum subsection: Peri-genual R
	9	59	20	-35	61	0.995	Corticospinal Tract R
	10	60	-42	-28	31	0.991	Arcuate Fasciculus L
							Superior Longitudinal Fasciculus 2 L

						Superior Longitudinal Fasciculus 3 L
11	62	43	-10	27	0.989	Arcuate Fasciculus R
						Superior Longitudinal Fasciculus 3 R
12	63	-42	-53	43	0.998	Superior Longitudinal Fasciculus 2 L
						Superior Longitudinal Fasciculus 3 L
13	65	19	15	4	0.99	Anterior Thalamic Radiation R
14	69	-19	4	-12	0.998	Inferior Fronto-Occipital Fasciculus L
						Uncinate Fasciculus L
15	69	-43	-10	-35	0.994	Inferior Longitudinal Fasciculus L
16	69	11	49	36	0.994	Anterior Thalamic Radiation R
17	70	-8	40	-19	0.993	Cingulum subsection: Dorsal L
						Uncinate Fasciculus L
18	71	33	10	49	0.996	Superior Longitudinal Fasciculus 2 R
19	72	8	-35	41	0.992	Cingulum subsection: Dorsal R
20	72	47	26	10	0.995	Frontal Aslant Tract R
						Superior Longitudinal Fasciculus 3 R
21	73	29	27	-12	0.996	Uncinate Fasciculus R
22	74	-21	-57	54	0.999	Superior Longitudinal Fasciculus 1 L
23	76	32	-43	50	0.998	Superior Longitudinal Fasciculus 1 R
24	81	10	-83	29	0.994	Forceps Major
25	88	-44	-59	34	0.998	Superior Longitudinal Fasciculus 2 L
						Superior Longitudinal Fasciculus 3 L
26	92	-28	-61	42	0.995	Superior Longitudinal Fasciculus 1 L
						Superior Longitudinal Fasciculus 2 L
27	102	-44	6	14	0.995	Arcuate Fasciculus L
						Frontal Aslant Tract L
						Superior Longitudinal Fasciculus 3 L
28	106	-26	-71	1	0.991	Forceps Major
						Inferior Fronto-Occipital Fasciculus L

						Middle Longitudinal Fasciculus L
						Optic Radiation L
						Vertical Occipital Fasciculus L
29	109	34	-63	9	0.992	Forceps Major
						Inferior Fronto-Occipital Fasciculus R
						Middle Longitudinal Fasciculus R
						Optic Radiation R
30	111	-48	15	12	0.997	Arcuate Fasciculus L
						Frontal Aslant Tract L
						Superior Longitudinal Fasciculus 3 L
31	115	-36	31	21	0.997	Superior Longitudinal Fasciculus 2 L
32	115	60	-41	-2	0.995	Arcuate Fasciculus R
						Inferior Longitudinal Fasciculus R
33	115	20	-69	13	0.995	Forceps Major
34	118	-19	-82	30	0.994	Forceps Major
						Middle Longitudinal Fasciculus L
						Optic Radiation L
						Vertical Occipital Fasciculus L
35	119	49	-56	-8	0.997	Inferior Longitudinal Fasciculus R
36	128	39	30	-5	0.995	Anterior Thalamic Radiation R
						Forceps Minor
						Inferior Fronto-Occipital Fasciculus R
						Superior Longitudinal Fasciculus 3 R
37	129	12	-49	13	0.996	Cingulum subsection: Dorsal R
						Forceps Major
38	138	-51	-37	-18	0.997	Arcuate Fasciculus L
						Inferior Fronto-Occipital Fasciculus L
						Inferior Longitudinal Fasciculus L
39	138	-8	-47	28	0.994	Cingulum subsection: Dorsal L

40	143	47	14	8	0.992	Arcuate Fasciculus R
						Frontal Aslant Tract R
						Superior Longitudinal Fasciculus 3 R
41	149	-10	-3	64	0.996	Frontal Aslant Tract L
						Superior Longitudinal Fasciculus 1 L
						Superior Thalamic Radiation L
42	167	-28	-62	12	0.994	Forceps Major
						Inferior Fronto-Occipital Fasciculus L
						Middle Longitudinal Fasciculus L
						Optic Radiation L
						Vertical Occipital Fasciculus L
43	178	-42	-25	10	0.997	Acoustic Radiation L
						Middle Longitudinal Fasciculus L
44	192	42	-43	32	0.999	Arcuate Fasciculus R
						Superior Longitudinal Fasciculus 2 R
						Superior Longitudinal Fasciculus 3 R
45	193	-7	23	23	0.995	Cingulum subsection: Dorsal L
						Cingulum subsection: Peri-genual L
46	196	39	20	35	0.995	Superior Longitudinal Fasciculus 2 R
47	212	-13	12	-4	0.998	Anterior Thalamic Radiation L
						Inferior Fronto-Occipital Fasciculus L
48	239	13	9	57	0.994	Frontal Aslant Tract R
						Superior Longitudinal Fasciculus 1 R
						Superior Thalamic Radiation R
49	247	50	-17	23	0.994	Arcuate Fasciculus R
						Superior Longitudinal Fasciculus 3 R
50	258	-7	-30	-15	1	Acoustic Radiation L
						Corticospinal Tract L
						Optic Radiation L

						Superior Thalamic Radiation L
51	278	-33	24	-14	0.997	Anterior Thalamic Radiation L
						Cingulum subsection: Dorsal L
						Inferior Fronto-Occipital Fasciculus L
						Uncinate Fasciculus L
52	279	44	-74	9	0.999	Forceps Major
						Vertical Occipital Fasciculus R
53	290	-49	-37	43	0.998	Arcuate Fasciculus L
						Superior Longitudinal Fasciculus 2 L
						Superior Longitudinal Fasciculus 3 L
54	321	-49	-4	-27	0.994	Fornix L
						Inferior Longitudinal Fasciculus L
						Middle Longitudinal Fasciculus L
						Uncinate Fasciculus L
55	322	-52	-50	25	0.999	Arcuate Fasciculus L
						Acoustic Radiation L
						Middle Longitudinal Fasciculus L
						Superior Longitudinal Fasciculus 2 L
						Superior Longitudinal Fasciculus 3 L
56	337	-12	-93	6	0.995	Forceps Major
						Middle Longitudinal Fasciculus L
						Optic Radiation L
						Vertical Occipital Fasciculus L
57	353	-38	-7	51	0.996	Corticospinal Tract L
						Superior Longitudinal Fasciculus 1 L
						Superior Longitudinal Fasciculus 2 L
						Superior Thalamic Radiation L
58	356	14	55	18	0.997	Anterior Thalamic Radiation R
						Cingulum subsection: Dorsal R

						Forceps Minor
						Inferior Fronto-Occipital Fasciculus R
						Uncinate Fasciculus R
59	360	-43	-24	50	0.998	Corticospinal Tract L
						Superior Longitudinal Fasciculus 2 L
						Superior Longitudinal Fasciculus 3 L
60	386	-52	-15	37	0.996	Arcuate Fasciculus L
						Superior Longitudinal Fasciculus 2 L
						Superior Longitudinal Fasciculus 3 L
61	449	-31	16	46	0.998	Arcuate Fasciculus L
						Superior Longitudinal Fasciculus 2 L
						Superior Longitudinal Fasciculus 3 L
62	468	-6	-71	34	0.996	Superior Longitudinal Fasciculus 1 L
63	475	-54	-12	-20	0.996	Arcuate Fasciculus L
						Inferior Longitudinal Fasciculus L
64	478	-7	-36	63	0.998	Corticospinal Tract L
						Superior Longitudinal Fasciculus 1 L
65	574	-35	-84	1	0.998	Forceps Major
						Inferior Fronto-Occipital Fasciculus L
						Inferior Longitudinal Fasciculus L
						Middle Longitudinal Fasciculus L
						Optic Radiation L
						Vertical Occipital Fasciculus L
66	1036	-14	56	12	0.996	Anterior Thalamic Radiation L
						Cingulum subsection: Dorsal L
						Frontal Aslant Tract L
						Forceps Minor
						Inferior Fronto-Occipital Fasciculus L
						Superior Longitudinal Fasciculus 1 L

					Superior Thalamic Radiation L
					Uncinate Fasciculus L
	67	1086	1 -14 19	0.999	Anterior Commissure
					Acoustic Radiation L
					Anterior Thalamic Radiation L
					Anterior Thalamic Radiation R
					Fornix L
					Fornix R
					Optic Radiation L
					Superior Thalamic Radiation L
					Superior Thalamic Radiation R
ICVF					
	1	64	-20 -44 54	0.995	Corticospinal Tract L
					Superior Longitudinal Fasciculus 1 L
	2	66	-56 -20 -15	0.994	Arcuate Fasciculus L
					Inferior Longitudinal Fasciculus L
	3	73	14 -72 17	0.998	Forceps Major
	4	77	-53 -28 -24	0.997	Inferior Longitudinal Fasciculus L

Additional Table 1 B TBSS Analysis of White Matter Fiber Tracts Between HC and NPSLE

Metrics	Cluster index	Number of voxels	Signal peaks (x,y,z)	Maximally 1-p value	White matter tracts
FA					
	1	62	-41 -74 10	0.999	Vertical Occipital Fasciculus L
	2	141	-23 -58 -52	0.999	Middle Cerebellar Peduncle
	3	3751	-27 -52 -52	1	Corticospinal Tract L
					Middle Cerebellar Peduncle

4	4574	9	-48	-59	1	Corticospinal Tract R
						Middle Cerebellar Peduncle
5	81098	36	-6	-37	1	Anterior Commissure
						Arcuate Fasciculus L
						Arcuate Fasciculus R
						Acoustic Radiation L
						Acoustic Radiation R
						Anterior Thalamic Radiation L
						Anterior Thalamic Radiation R
						Cingulum subsection: Dorsal L
						Cingulum subsection: Dorsal R
						Cingulum subsection: Peri-genual L
						Cingulum subsection: Peri-genual R
						Cingulum subsection: Temporal L
						Cingulum subsection: Temporal R
						Corticospinal Tract L
						Corticospinal Tract R
						Frontal Aslant Tract L
						Frontal Aslant Tract R
						Forceps Major
						Forceps Minor
						Fornix L
						Fornix R
						Inferior Fronto-Occipital Fasciculus L
						Inferior Fronto-Occipital Fasciculus R
						Inferior Longitudinal Fasciculus L
						Inferior Longitudinal Fasciculus R
						Middle Cerebellar Peduncle
						Middle Longitudinal Fasciculus L

							Middle Longitudinal Fasciculus R
							Optic Radiation L
							Optic Radiation R
							Superior Longitudinal Fasciculus 1 L
							Superior Longitudinal Fasciculus 1 R
							Superior Longitudinal Fasciculus 2 L
							Superior Longitudinal Fasciculus 2 R
							Superior Longitudinal Fasciculus 3 L
							Superior Longitudinal Fasciculus 3 R
							Superior Thalamic Radiation L
							Superior Thalamic Radiation R
							Uncinate Fasciculus L
							Uncinate Fasciculus R
							Vertical Occipital Fasciculus L
							Vertical Occipital Fasciculus R
RD							
	1	50	19	13	57	0.996	Frontal Aslant Tract R:12.2407
							Superior Longitudinal Fasciculus 1 R
	2	51	25	-2	44	0.988	Superior Longitudinal Fasciculus 2 R
							Superior Thalamic Radiation R
	3	58	19	-6	61	0.997	Superior Longitudinal Fasciculus 1 R
							Superior Thalamic Radiation R
	4	59	-43	-6	43	0.993	Superior Longitudinal Fasciculus 2 L
	5	69	-10	-21	69	0.992	Corticospinal Tract L
							Superior Thalamic Radiation L
	6	81	-13	-84	33	0.996	Forceps Major
							Middle Longitudinal Fasciculus L
							Optic Radiation L
							Vertical Occipital Fasciculus L

7	81	19	-35	67	0.997	Corticospinal Tract R
						Superior Longitudinal Fasciculus 1 R
8	84	17	-34	-1	0.999	Acoustic Radiation R
						Optic Radiation R
						Superior Thalamic Radiation R
9	87	46	-39	45	0.998	Arcuate Fasciculus R
						Superior Longitudinal Fasciculus 3 R
10	89	-35	-7	-19	0.993	Fornix L
						Inferior Fronto-Occipital Fasciculus L
						Inferior Longitudinal Fasciculus L
						Uncinate Fasciculus L
11	96	-25	-81	6	0.993	Forceps Major
						Inferior Fronto-Occipital Fasciculus L
						Middle Longitudinal Fasciculus L
						Optic Radiation L
						Vertical Occipital Fasciculus L
12	100	-31	-14	53	0.993	Corticospinal Tract L
						Superior Thalamic Radiation L
13	109	38	-63	41	0.997	Inferior Fronto-Occipital Fasciculus R
						Superior Longitudinal Fasciculus 2 R
						Superior Longitudinal Fasciculus 3 R
14	116	-32	-43	35	0.992	Superior Longitudinal Fasciculus 2 L
						Superior Longitudinal Fasciculus 3 L
15	121	-46	-56	33	0.997	Superior Longitudinal Fasciculus 2 L
						Superior Longitudinal Fasciculus 3 L
16	144	-59	-28	-10	0.997	Inferior Longitudinal Fasciculus L
17	144	-38	34	7	0.994	Arcuate Fasciculus L
						Anterior Thalamic Radiation L
						Superior Longitudinal Fasciculus 3 L

18	148	17	15	-23	0.999	Uncinate Fasciculus R
19	149	-17	-8	50	0.993	Corticospinal Tract L
						Superior Longitudinal Fasciculus 1 L
						Superior Thalamic Radiation L
20	164	-48	-19	7	0.997	Acoustic Radiation L
						Middle Longitudinal Fasciculus L
21	179	-50	-36	40	0.997	Arcuate Fasciculus L
						Superior Longitudinal Fasciculus 3 L
22	198	39	6	43	0.999	Frontal Aslant Tract R
						Superior Longitudinal Fasciculus 2 R
23	207	12	-23	64	0.993	Corticospinal Tract R
						Superior Thalamic Radiation R
24	217	60	-24	-11	0.997	Inferior Fronto-Occipital Fasciculus R
						Inferior Longitudinal Fasciculus R
						Optic Radiation R
25	231	-34	-28	38	0.991	Arcuate Fasciculus L
						Superior Longitudinal Fasciculus 2 L
						Superior Longitudinal Fasciculus 3 L
26	232	-7	52	-18	0.996	Anterior Thalamic Radiation L
						Cingulum subsection: Dorsal L
						Forceps Minor
						Inferior Fronto-Occipital Fasciculus L
						Uncinate Fasciculus L
27	242	37	17	40	0.999	Superior Longitudinal Fasciculus 2 R
28	263	16	11	4	0.995	Anterior Thalamic Radiation R
						Inferior Fronto-Occipital Fasciculus R
29	318	-24	-34	63	0.997	Corticospinal Tract L
						Superior Longitudinal Fasciculus 1 L
30	446	43	-3	43	0.994	Arcuate Fasciculus R

						Frontal Aslant Tract R
						Superior Longitudinal Fasciculus 2 R
						Superior Longitudinal Fasciculus 3 R
31	455	-14	11	1	0.996	Anterior Thalamic Radiation L
						Inferior Fronto-Occipital Fasciculus L
						Uncinate Fasciculus L
32	457	-46	14	12	0.995	Arcuate Fasciculus L
						Frontal Aslant Tract L
						Superior Longitudinal Fasciculus 3 L
33	580	-51	-53	-7	0.997	Arcuate Fasciculus L
						Inferior Fronto-Occipital Fasciculus L
						Inferior Longitudinal Fasciculus L
						Optic Radiation L
34	730	-40	17	36	0.999	Anterior Thalamic Radiation L
						Frontal Aslant Tract L
						Forceps Minor
						Inferior Fronto-Occipital Fasciculus L
						Superior Longitudinal Fasciculus 2 L
35	2954	-5	7	-11	1	Anterior Commissure
						Acoustic Radiation L
						Acoustic Radiation R
						Anterior Thalamic Radiation L
						Anterior Thalamic Radiation R
						Cingulum subsection: Dorsal L
						Cingulum subsection: Dorsal R
						Cingulum subsection: Temporal L
						Cingulum subsection: Temporal R
						Corticospinal Tract R
						Forceps Major

						Fornix L
						Fornix R
						Optic Radiation L
						Optic Radiation R
						Superior Thalamic Radiation L
						Superior Thalamic Radiation R
36	20892	24	-47	5	0.999	Anterior Commissure
						Arcuate Fasciculus L
						Arcuate Fasciculus R
						Acoustic Radiation L
						Acoustic Radiation R
						Anterior Thalamic Radiation L
						Anterior Thalamic Radiation R
						Cingulum subsection: Dorsal L
						Cingulum subsection: Dorsal R
						Cingulum subsection: Peri-genual L
						Cingulum subsection: Peri-genual R
						Cingulum subsection: Temporal R
						Corticospinal Tract L
						Corticospinal Tract R
						Frontal Aslant Tract L
						Frontal Aslant Tract R
						Forceps Major
						Forceps Minor
						Inferior Fronto-Occipital Fasciculus L
						Inferior Fronto-Occipital Fasciculus R
						Inferior Longitudinal Fasciculus L
						Inferior Longitudinal Fasciculus R
						Middle Longitudinal Fasciculus L

							Middle Longitudinal Fasciculus R
							Optic Radiation L
							Optic Radiation R
							Superior Longitudinal Fasciculus 1 L
							Superior Longitudinal Fasciculus 1 R
							Superior Longitudinal Fasciculus 2 L
							Superior Longitudinal Fasciculus 2 R
							Superior Longitudinal Fasciculus 3 L
							Superior Longitudinal Fasciculus 3 R
							Superior Thalamic Radiation L
							Superior Thalamic Radiation R
							Uncinate Fasciculus L
							Uncinate Fasciculus R
							Vertical Occipital Fasciculus L
							Vertical Occipital Fasciculus R
AK							
	1	50	-14	-16	58	0.99	Corticospinal Tract L
							Superior Longitudinal Fasciculus 1 L
							Superior Thalamic Radiation L
	2	50	-21	-90	17	0.993	Forceps Major
							Middle Longitudinal Fasciculus L
							Optic Radiation L
							Vertical Occipital Fasciculus L
	3	53	58	-36	21	0.993	Arcuate Fasciculus R
	4	53	24	-26	-20	0.996	Cingulum subsection: Temporal R
	5	57	33	-15	7	0.992	Middle Longitudinal Fasciculus R
	6	57	-25	-89	0	0.996	Forceps Major
							Inferior Fronto-Occipital Fasciculus L
							Optic Radiation L

						Vertical Occipital Fasciculus L
7	61	25	-2	44	0.994	Superior Longitudinal Fasciculus 2 R
						Superior Thalamic Radiation R
8	63	31	3	53	0.992	Superior Longitudinal Fasciculus 2 R
9	64	-23	-24	-22	0.994	Cingulum subsection: Temporal L
10	65	-26	11	20	0.991	Frontal Aslant Tract L
						Superior Thalamic Radiation L
11	66	57	-34	-7	0.993	Arcuate Fasciculus R
						Inferior Longitudinal Fasciculus R:
12	68	32	-1	36	0.993	Arcuate Fasciculus R
						Superior Longitudinal Fasciculus 2 R
						Superior Longitudinal Fasciculus 3 R
13	71	-27	-39	32	1	Middle Longitudinal Fasciculus L
						Superior Longitudinal Fasciculus 2 L
14	74	-31	5	6	0.994	Inferior Fronto-Occipital Fasciculus L
15	82	-36	23	-12	0.993	Inferior Fronto-Occipital Fasciculus L
16	83	-36	38	19	0.993	Forceps Minor
						Superior Longitudinal Fasciculus 2 L
17	85	15	-18	59	0.993	Corticospinal Tract R
						Superior Longitudinal Fasciculus 1 R
						Superior Thalamic Radiation R
18	89	25	-5	34	0.992	Corticospinal Tract R
						Superior Thalamic Radiation R
19	94	35	-43	21	0.996	Arcuate Fasciculus R
						Inferior Fronto-Occipital Fasciculus R
						Middle Longitudinal Fasciculus R
						Optic Radiation R
20	95	-22	-64	-46	0.994	Middle Cerebellar Peduncle
21	96	12	-29	58	0.993	Corticospinal Tract R

						Superior Longitudinal Fasciculus 1 R
22	110	-26	-21	-5	0.994	Arcuate Fasciculus R
						Corticospinal Tract R
						Frontal Aslant Tract R
						Superior Longitudinal Fasciculus 3 R
23	110	29	3	18	0.994	Acoustic Radiation L
						Corticospinal Tract L
						Fornix L:0.8806
						Optic Radiation L
24	121	19	-18	62	0.998	Corticospinal Tract R
						Superior Thalamic Radiation R
25	126	-5	-45	-25	0.999	Corticospinal Tract L
26	136	32	-3	26	0.999	Arcuate Fasciculus R
						Corticospinal Tract R
						Frontal Aslant Tract R
						Superior Longitudinal Fasciculus 2 R
						Superior Longitudinal Fasciculus 3 R
						Superior Thalamic Radiation R
27	136	50	-51	-3	0.995	Inferior Longitudinal Fasciculus R
28	136	-7	-85	24	0.997	Forceps Major
						Middle Longitudinal Fasciculus L
						Optic Radiation L
29	140	34	-24	25	0.995	Arcuate Fasciculus R
						Superior Longitudinal Fasciculus 2 R
						Superior Longitudinal Fasciculus 3 R
30	145	17	-2	9	0.993	Anterior Thalamic Radiation R
						Corticospinal Tract R
						Superior Thalamic Radiation R
31	146	-25	-35	-11	0.998	Cingulum subsection: Temporal L

32	148	-40	-11	-37	0.995	Cingulum subsection: Temporal L
						Fornix L
						Inferior Longitudinal Fasciculus L
33	152	-22	53	-9	0.993	Anterior Thalamic Radiation L
						Forceps Minor
						Inferior Fronto-Occipital Fasciculus L
						Uncinate Fasciculus L
34	164	18	30	49	1	Frontal Aslant Tract R
						Superior Longitudinal Fasciculus 1 R
						Superior Thalamic Radiation R
35	172	-23	-15	7	0.993	Anterior Thalamic Radiation L
						Corticospinal Tract L
						Superior Thalamic Radiation L
36	180	-14	1	8	0.993	Anterior Thalamic Radiation L
						Inferior Fronto-Occipital Fasciculus L
37	181	40	-74	7	0.996	Forceps Major
						Vertical Occipital Fasciculus R
38	186	-20	4	-12	0.998	Inferior Fronto-Occipital Fasciculus L
						Uncinate Fasciculus L
39	212	-41	19	33	0.996	Superior Longitudinal Fasciculus 2 L
40	216	-11	60	-4	0.996	Anterior Thalamic Radiation L
						Cingulum subsection: Dorsal L
						Forceps Minor
						Inferior Fronto-Occipital Fasciculus L
						Uncinate Fasciculus L
41	231	-34	-6	-6	0.994	Anterior Commissure
						Acoustic Radiation L
						Inferior Fronto-Occipital Fasciculus L
						Inferior Longitudinal Fasciculus L

						Middle Longitudinal Fasciculus L
						Optic Radiation L
						Uncinate Fasciculus L
42	239	-15	27	41	0.994	Anterior Thalamic Radiation L
						Forceps Minor
						Superior Longitudinal Fasciculus 1 L
43	261	-21	-10	36	0.999	Arcuate Fasciculus L
						Corticospinal Tract L
						Frontal Aslant Tract L
						Superior Longitudinal Fasciculus 2 L
						Superior Thalamic Radiation L
44	275	20	-70	14	0.998	Forceps Major
						Middle Longitudinal Fasciculus R
						Optic Radiation R
						Vertical Occipital Fasciculus R
45	280	-25	-18	47	0.995	Corticospinal Tract L
						Superior Longitudinal Fasciculus 1 L
						Superior Longitudinal Fasciculus 2 L
						Superior Thalamic Radiation L
46	295	-50	5	-12	0.997	Acoustic Radiation L
						Fornix L
						Inferior Longitudinal Fasciculus L
						Middle Longitudinal Fasciculus L
						Uncinate Fasciculus L
47	306	12	60	12	0.998	Anterior Thalamic Radiation R
						Cingulum subsection: Dorsal R
						Forceps Minor
						Inferior Fronto-Occipital Fasciculus R
						Uncinate Fasciculus R

48	388	-8	-42	31	0.999	Cingulum subsection: Dorsal L
						Forceps Major
49	403	0	-3	-15	1	Anterior Commissure
						Fornix L
						Fornix R
50	453	14	-64	2	0.999	Forceps Major
						Inferior Fronto-Occipital Fasciculus R
						Optic Radiation R
51	759	9	14	28	1	Cingulum subsection: Dorsal R
						Cingulum subsection: Peri-genual R
52	1004	-3	-46	-66	1	Corticospinal Tract L
						Corticospinal Tract R
						Middle Cerebellar Peduncle
53	2855	-7	-33	-12	1	Anterior Commissure
						Acoustic Radiation L
						Acoustic Radiation R
						Anterior Thalamic Radiation L
						Anterior Thalamic Radiation R
						Cingulum subsection: Dorsal R
						Cingulum subsection: Temporal L
						Cingulum subsection: Temporal R
						Corticospinal Tract L
						Corticospinal Tract R
						Forceps Major
						Fornix L
						Fornix R
						Optic Radiation L
						Optic Radiation R
						Superior Thalamic Radiation L

							Superior Thalamic Radiation R
	54	8292	-57	-47	2	1	Anterior Commissure
							Arcuate Fasciculus L
							Acoustic Radiation L
							Cingulum subsection: Dorsal L
							Corticospinal Tract L
							Forceps Major
							Inferior Fronto-Occipital Fasciculus L
							Inferior Longitudinal Fasciculus L
							Middle Longitudinal Fasciculus L
							Optic Radiation L
							Superior Longitudinal Fasciculus 1 L
							Superior Longitudinal Fasciculus 2 L
							Superior Longitudinal Fasciculus 3 L
							Vertical Occipital Fasciculus L
RK							
	1	66	20	-69	13	0.996	Forceps Major
	2	102	-9	-26	2	0.998	Acoustic Radiation L
							Superior Thalamic Radiation L
	3	278	17	-20	-11	0.998	Acoustic Radiation R
							Corticospinal Tract R
							Optic Radiation R
							Superior Thalamic Radiation R
	4	63799	-1	12	-29	1	Anterior Commissure
							Arcuate Fasciculus L
							Arcuate Fasciculus R
							Acoustic Radiation L
							Acoustic Radiation R
							Anterior Thalamic Radiation L

Anterior Thalamic Radiation R

Cingulum subsection: Dorsal L

Cingulum subsection: Dorsal R

Cingulum subsection: Peri-genual L

Cingulum subsection: Peri-genual R

Cingulum subsection: Temporal L

Cingulum subsection: Temporal R

Corticospinal Tract L

Corticospinal Tract R

Frontal Aslant Tract L

Frontal Aslant Tract R

Forceps Major

Forceps Minor

Fornix L

Fornix R

Inferior Fronto-Occipital Fasciculus L

Inferior Fronto-Occipital Fasciculus R

Inferior Longitudinal Fasciculus L

Inferior Longitudinal Fasciculus R

Middle Longitudinal Fasciculus L

Middle Longitudinal Fasciculus R

Optic Radiation L

Optic Radiation R

Superior Longitudinal Fasciculus 1 L

Superior Longitudinal Fasciculus 1 R

Superior Longitudinal Fasciculus 2 L

Superior Longitudinal Fasciculus 2 R

Superior Longitudinal Fasciculus 3 L

Superior Longitudinal Fasciculus 3 R

							Superior Thalamic Radiation L
							Superior Thalamic Radiation R
							Uncinate Fasciculus L
							Uncinate Fasciculus R
							Vertical Occipital Fasciculus L
							Vertical Occipital Fasciculus R
KFA							
	1	54	-45	8	-21	0.995	Middle Longitudinal Fasciculus L
							Uncinate Fasciculus L
	2	56	53	-53	9	0.999	Arcuate Fasciculus R
	3	336	10	38	4	1	Anterior Thalamic Radiation R
							Cingulum subsection: Dorsal R
							Cingulum subsection: Peri-genual R
	4	773	42	-56	-38	0.998	Middle Cerebellar Peduncle
	5	3216	30	-49	-49	1	Middle Cerebellar Peduncle
	6	3747	-18	-65	-49	1	Middle Cerebellar Peduncle
	7	75722	-47	-16	-32	1	Anterior Commissure
							Arcuate Fasciculus L
							Arcuate Fasciculus R
							Acoustic Radiation L
							Acoustic Radiation R
							Anterior Thalamic Radiation L
							Anterior Thalamic Radiation R
							Cingulum subsection: Dorsal L
							Cingulum subsection: Dorsal R
							Cingulum subsection: Peri-genual L
							Cingulum subsection: Peri-genual R
							Cingulum subsection: Temporal L
							Cingulum subsection: Temporal R

Corticospinal Tract L

Corticospinal Tract R

Frontal Aslant Tract L

Frontal Aslant Tract R

Forceps Major

Forceps Minor

Fornix L

Fornix R

Inferior Fronto-Occipital Fasciculus L

Inferior Fronto-Occipital Fasciculus R

Inferior Longitudinal Fasciculus L

Inferior Longitudinal Fasciculus R

Middle Longitudinal Fasciculus L

Middle Longitudinal Fasciculus R

Optic Radiation L

Optic Radiation R

Superior Longitudinal Fasciculus 1 L

Superior Longitudinal Fasciculus 1 R

Superior Longitudinal Fasciculus 2 L

Superior Longitudinal Fasciculus 2 R

Superior Longitudinal Fasciculus 3 L

Superior Longitudinal Fasciculus 3 R

Superior Thalamic Radiation L

Superior Thalamic Radiation R

Uncinate Fasciculus L

Uncinate Fasciculus R

Vertical Occipital Fasciculus L

Vertical Occipital Fasciculus R

MK

1	69838	27	-3	-35	1	Superior Longitudinal Fasciculus 1 R
						Superior Thalamic Radiation R
2	60	21	-4	62	0.996	Anterior Commissure
						Arcuate Fasciculus L
						Arcuate Fasciculus R
						Acoustic Radiation L
						Acoustic Radiation R
						Anterior Thalamic Radiation L
						Anterior Thalamic Radiation R
						Cingulum subsection: Dorsal L
						Cingulum subsection: Dorsal R
						Cingulum subsection: Peri-genual L
						Cingulum subsection: Peri-genual R
						Cingulum subsection: Temporal L
						Cingulum subsection: Temporal R
						Corticospinal Tract L
						Corticospinal Tract R
						Frontal Aslant Tract L
						Frontal Aslant Tract R
						Forceps Major
						Forceps Minor
						Fornix L
						Fornix R
						Inferior Fronto-Occipital Fasciculus L
						Inferior Fronto-Occipital Fasciculus R
						Inferior Longitudinal Fasciculus L
						Inferior Longitudinal Fasciculus R
						Middle Longitudinal Fasciculus L
						Middle Longitudinal Fasciculus R

							Optic Radiation L
							Optic Radiation R
							Superior Longitudinal Fasciculus 1 L
							Superior Longitudinal Fasciculus 1 R
							Superior Longitudinal Fasciculus 2 L
							Superior Longitudinal Fasciculus 2 R
							Superior Longitudinal Fasciculus 3 L
							Superior Longitudinal Fasciculus 3 R
							Superior Thalamic Radiation L
							Superior Thalamic Radiation R
							Uncinate Fasciculus L
							Uncinate Fasciculus R
							Vertical Occipital Fasciculus L
							Vertical Occipital Fasciculus R
GA							
	1	66	-7	-30	-41	0.998	Corticospinal Tract L
							Middle Cerebellar Peduncle
	2	243	38	-49	-36	0.996	Middle Cerebellar Peduncle
	3	481	21	-41	-27	0.999	Middle Cerebellar Peduncle
	4	1130	14	-62	-52	0.999	Middle Cerebellar Peduncle
	5	1689	-23	-58	-52	0.999	Middle Cerebellar Peduncle
	6	79473	-34	-6	-39	1	Anterior Commissure
							Arcuate Fasciculus L
							Arcuate Fasciculus R
							Acoustic Radiation L
							Acoustic Radiation R
							Anterior Thalamic Radiation L
							Anterior Thalamic Radiation R

Cingulum subsection: Dorsal L

Cingulum subsection: Dorsal R

Cingulum subsection: Peri-genual L

Cingulum subsection: Peri-genual R

Cingulum subsection: Temporal L

Cingulum subsection: Temporal R

Corticospinal Tract L

Corticospinal Tract R

Frontal Aslant Tract L

Frontal Aslant Tract R

Forceps Major

Forceps Minor

Fornix L

Fornix R

Inferior Fronto-Occipital Fasciculus L

Inferior Fronto-Occipital Fasciculus R

Inferior Longitudinal Fasciculus L

Inferior Longitudinal Fasciculus R

Middle Longitudinal Fasciculus L

Middle Longitudinal Fasciculus R

Optic Radiation L

Optic Radiation R

Superior Longitudinal Fasciculus 1 L

Superior Longitudinal Fasciculus 1 R

Superior Longitudinal Fasciculus 2 L

Superior Longitudinal Fasciculus 2 R

Superior Longitudinal Fasciculus 3 L

Superior Longitudinal Fasciculus 3 R

Superior Thalamic Radiation L

							Superior Thalamic Radiation R
							Uncinate Fasciculus L
							Uncinate Fasciculus R
							Vertical Occipital Fasciculus L
							Vertical Occipital Fasciculus R
NG							
	1	68	10	38	4	0.989	Cingulum subsection: Dorsal R
							Cingulum subsection: Peri-genual R
	2	70	-47	-53	30	0.997	Superior Longitudinal Fasciculus 2 L
MSD							
	1	58	31	9	17	0.993	Arcuate Fasciculus R
							Frontal Aslant Tract R
							Superior Longitudinal Fasciculus 3 R
	2	86	-29	-55	14	0.995	Forceps Major
							Inferior Fronto-Occipital Fasciculus L
							Middle Longitudinal Fasciculus L
							Optic Radiation L
	3	159	31	-2	39	0.992	Superior Longitudinal Fasciculus 2 R
							Superior Longitudinal Fasciculus 3 R
							Superior Thalamic Radiation R
	4	226	26	-48	0	0.999	Cingulum subsection: Temporal R
							Forceps Major
							Inferior Fronto-Occipital Fasciculus R
							Inferior Longitudinal Fasciculus R
							Optic Radiation R
	5	646	5	-17	26	0.996	Cingulum subsection: Dorsal L
							Cingulum subsection: Dorsal R
	6	2539	1	1	-14	0.999	Anterior Commissure
							Acoustic Radiation L

						Acoustic Radiation R
						Anterior Thalamic Radiation L
						Anterior Thalamic Radiation R
						Cingulum subsection: Dorsal L
						Cingulum subsection: Dorsal R
						Cingulum subsection: Temporal L
						Cingulum subsection: Temporal R
						Corticospinal Tract R
						Forceps Major
						Fornix L
						Fornix R
						Optic Radiation L
						Optic Radiation R
						Superior Thalamic Radiation L
						Superior Thalamic Radiation R
RTOP						
1	111	-16	10	56	0.999	Frontal Aslant Tract L
						Superior Longitudinal Fasciculus 1 L
						Superior Thalamic Radiation L
2	120	-18	-9	-10	0.999	Anterior Commissure
						Anterior Thalamic Radiation L
						Corticospinal Tract L
3	108074	-29	-57	-50	1	Anterior Commissure
						Arcuate Fasciculus L
						Arcuate Fasciculus R
						Acoustic Radiation L
						Acoustic Radiation R
						Anterior Thalamic Radiation L
						Anterior Thalamic Radiation R

Cingulum subsection: Dorsal L

Cingulum subsection: Dorsal R

Cingulum subsection: Peri-genual L

Cingulum subsection: Peri-genual R

Cingulum subsection: Temporal L

Cingulum subsection: Temporal R

Corticospinal Tract L

Corticospinal Tract R

Frontal Aslant Tract L

Frontal Aslant Tract R

Forceps Major

Forceps Minor

Fornix L

Fornix R

Inferior Fronto-Occipital Fasciculus L

Inferior Fronto-Occipital Fasciculus R

Inferior Longitudinal Fasciculus L

Inferior Longitudinal Fasciculus R

Middle Cerebellar Peduncle

Middle Longitudinal Fasciculus L

Middle Longitudinal Fasciculus R

Optic Radiation L

Optic Radiation R

Superior Longitudinal Fasciculus 1 L

Superior Longitudinal Fasciculus 1 R

Superior Longitudinal Fasciculus 2 L

Superior Longitudinal Fasciculus 2 R

Superior Longitudinal Fasciculus 3 L

Superior Longitudinal Fasciculus 3 R

						Superior Thalamic Radiation L
						Superior Thalamic Radiation R
						Uncinate Fasciculus L
						Uncinate Fasciculus R
						Vertical Occipital Fasciculus L
						Vertical Occipital Fasciculus R
RTAP						
1	114	-16	18	52	0.999	Frontal Aslant Tract L
						Superior Longitudinal Fasciculus 1 L
						Superior Thalamic Radiation L
2	108302	-29	-55	-49	1	Anterior Commissure
						Arcuate Fasciculus L
						Arcuate Fasciculus R
						Acoustic Radiation L
						Acoustic Radiation R
						Anterior Thalamic Radiation L
						Anterior Thalamic Radiation R
						Cingulum subsection: Dorsal L
						Cingulum subsection: Dorsal R
						Cingulum subsection: Peri-genual L
						Cingulum subsection: Peri-genual R
						Cingulum subsection: Temporal L
						Cingulum subsection: Temporal R
						Corticospinal Tract L
						Corticospinal Tract R
						Frontal Aslant Tract L
						Frontal Aslant Tract R
						Forceps Major
						Forceps Minor

							Fornix L
							Fornix R
							Inferior Fronto-Occipital Fasciculus L
							Inferior Fronto-Occipital Fasciculus R
							Inferior Longitudinal Fasciculus L
							Inferior Longitudinal Fasciculus R
							Middle Cerebellar Peduncle
							Middle Longitudinal Fasciculus L
							Middle Longitudinal Fasciculus R
							Optic Radiation L
							Optic Radiation R
							Superior Longitudinal Fasciculus 1 L
							Superior Longitudinal Fasciculus 1 R
							Superior Longitudinal Fasciculus 2 L
							Superior Longitudinal Fasciculus 2 R
							Superior Longitudinal Fasciculus 3 L
							Superior Longitudinal Fasciculus 3 R
							Superior Thalamic Radiation L
							Superior Thalamic Radiation R
							Uncinate Fasciculus L
							Uncinate Fasciculus R
							Vertical Occipital Fasciculus L
							Vertical Occipital Fasciculus R
ECVF							
	1	55	50	-23	29	0.995	Arcuate Fasciculus R
							Superior Longitudinal Fasciculus 3 R
	2	60	18	4	57	0.992	Frontal Aslant Tract R
							Superior Longitudinal Fasciculus 1 R
							Superior Thalamic Radiation R

3	60	-41	-53	6	0.992	Arcuate Fasciculus L
						Inferior Longitudinal Fasciculus L
4	66	-28	-22	34	0.99	Corticospinal Tract L
						Superior Longitudinal Fasciculus 1 L
						Superior Longitudinal Fasciculus 2 L
5	68	21	-9	19	0.994	Corticospinal Tract R
						Superior Thalamic Radiation R
6	69	37	-9	32	0.991	Arcuate Fasciculus R
						Superior Longitudinal Fasciculus 2 R
						Superior Longitudinal Fasciculus 3 R
7	69	30	3	18	0.995	Arcuate Fasciculus R
						Corticospinal Tract R
8	89	-45	-56	-4	0.998	Arcuate Fasciculus L
						Inferior Longitudinal Fasciculus L:
9	91	37	-54	24	0.99	Arcuate Fasciculus R
						Inferior Fronto-Occipital Fasciculus R
						Middle Longitudinal Fasciculus R
						Superior Longitudinal Fasciculus 2 R
						Superior Longitudinal Fasciculus 3 R
10	93	28	11	16	0.992	Arcuate Fasciculus R
						Frontal Aslant Tract R
						Inferior Fronto-Occipital Fasciculus R
11	99	46	-39	43	0.995	Arcuate Fasciculus R
						Superior Longitudinal Fasciculus 3 R
12	102	-24	-62	49	0.997	Superior Longitudinal Fasciculus 1 L
13	113	27	-2	30	0.993	Corticospinal Tract R
						Frontal Aslant Tract R
						Superior Thalamic Radiation R
14	121	-53	-37	-8	0.996	Arcuate Fasciculus L

						Inferior Longitudinal Fasciculus L
15	146	10	38	8	1	Cingulum subsection: Dorsal R
						Cingulum subsection: Peri-genual R
16	198	-33	47	4	0.993	Anterior Thalamic Radiation L
						Forceps Minor
						Inferior Fronto-Occipital Fasciculus L
						Uncinate Fasciculus L
17	207	9	-9	37	0.999	Cingulum subsection: Dorsal R
18	225	-53	-41	-19	0.996	Arcuate Fasciculus L
						Inferior Fronto-Occipital Fasciculus L
						Inferior Longitudinal Fasciculus L
						Optic Radiation L
19	238	-31	46	15	0.995	Forceps Minor
						Superior Longitudinal Fasciculus 2 L
20	238	-18	18	43	0.992	Frontal Aslant Tract L
						Superior Longitudinal Fasciculus 1 L
21	262	13	-48	11	0.998	Cingulum subsection: Dorsal R
						Forceps Major
22	281	-28	-17	19	0.994	Corticospinal Tract L
						Superior Thalamic Radiation L
23	406	35	-70	1	0.994	Arcuate Fasciculus R
						Forceps Major
						Inferior Fronto-Occipital Fasciculus R
						Inferior Longitudinal Fasciculus R
						Middle Longitudinal Fasciculus R
						Optic Radiation R
						Vertical Occipital Fasciculus R
24	603	-15	7	8	0.995	Anterior Thalamic Radiation L
						Inferior Fronto-Occipital Fasciculus L

						Uncinate Fasciculus L
25	1045	-56	-6	15	0.998	Arcuate Fasciculus L
						Frontal Aslant Tract L
						Superior Longitudinal Fasciculus 2 L
						Superior Longitudinal Fasciculus 3 L
						Superior Thalamic Radiation L
26	8281	-45	-56	32	0.998	Anterior Commissure
						Arcuate Fasciculus L
						Acoustic Radiation L
						Cingulum subsection: Dorsal L
						Cingulum subsection: Temporal L
						Corticospinal Tract L
						Forceps Major
						Inferior Fronto-Occipital Fasciculus L
						Inferior Longitudinal Fasciculus L
						Middle Longitudinal Fasciculus L
						Optic Radiation L
						Superior Longitudinal Fasciculus 1 L
						Superior Longitudinal Fasciculus 2 L
						Superior Longitudinal Fasciculus 3 L
						Superior Thalamic Radiation L
						Vertical Occipital Fasciculus L
27	8884	36	-40	40	0.999	Arcuate Fasciculus R
						Anterior Thalamic Radiation L
						Anterior Thalamic Radiation R
						Cingulum subsection: Dorsal L
						Cingulum subsection: Dorsal R
						Cingulum subsection: Peri-genual L
						Corticospinal Tract R

					Frontal Aslant Tract R
					Forceps Major
					Forceps Minor
					Inferior Fronto-Occipital Fasciculus L
					Inferior Fronto-Occipital Fasciculus R
					Middle Longitudinal Fasciculus R
					Optic Radiation R
					Superior Longitudinal Fasciculus 1 R
					Superior Longitudinal Fasciculus 2 L
					Superior Longitudinal Fasciculus 2 R
					Superior Longitudinal Fasciculus 3 R
					Superior Thalamic Radiation R
					Uncinate Fasciculus L
					Uncinate Fasciculus R
ICVF					
	1	55	-10 -55 -40	0.998	Middle Cerebellar Peduncle
	2	56	4 -17 -29	0.993	Corticospinal Tract L
					Corticospinal Tract R
					Middle Cerebellar Peduncle
	3	82	-15 -50 -49	0.994	Middle Cerebellar Peduncle
	4	150	18 -72 -46	0.993	Middle Cerebellar Peduncle
	5	198	20 -67 -51	0.996	Middle Cerebellar Peduncle
	6	737	-33 -48 -49	0.998	Middle Cerebellar Peduncle
	7	86159	-50 -24 -28	1	Anterior Commissure
					Arcuate Fasciculus L
					Arcuate Fasciculus R
					Acoustic Radiation L
					Acoustic Radiation R

Anterior Thalamic Radiation L

Anterior Thalamic Radiation R

Cingulum subsection: Dorsal L

Cingulum subsection: Dorsal R

Cingulum subsection: Peri-genual L

Cingulum subsection: Peri-genual R

Cingulum subsection: Temporal L

Cingulum subsection: Temporal R

Corticospinal Tract L

Corticospinal Tract R

Frontal Aslant Tract L

Frontal Aslant Tract R

Forceps Major

Forceps Minor

Fornix L

Fornix R

Inferior Fronto-Occipital Fasciculus L

Inferior Fronto-Occipital Fasciculus R

Inferior Longitudinal Fasciculus L

Inferior Longitudinal Fasciculus R

Middle Longitudinal Fasciculus L

Middle Longitudinal Fasciculus R

Optic Radiation L

Optic Radiation R

Superior Longitudinal Fasciculus 1 L

Superior Longitudinal Fasciculus 1 R

Superior Longitudinal Fasciculus 2 L

Superior Longitudinal Fasciculus 2 R

Superior Longitudinal Fasciculus 3 L

Superior Longitudinal Fasciculus 3 R
Superior Thalamic Radiation L
Superior Thalamic Radiation R
Uncinate Fasciculus L
Uncinate Fasciculus R
Vertical Occipital Fasciculus L
Vertical Occipital Fasciculus R
