# Supplementary material for: Detection of white matter microstructural changes in patients with systemic lupus erythematosus based on multiple diffusion models and related diffusion metrics
Source: Neural Regen Res. 2025 Nov 25;21(6):2467–74. doi: 10.4103/NRR.NRR-D-25-00730 (PMC13211794; doi:10.4103/NRR.NRR-D-25-00730)
Supplement: Supplementary file 2 [file NRR-21-2467_Suppl2.pdf]

Additional Table 2A The AUC of White Matter Fiber Tract Metrics Between HC and Non-NPSLE

Metrics	Brain region	AUC
GA		
	Inferior_Fronto_Occipital_Fasciculus_R	0.934
	Inferior_Longitudinal_Fasciculus_L	0.923
	Optic_Radiation_R	0.923
	Frontal_Aslant_Tract_L	0.901
	Vertical_Occipital_Fasciculus_L	0.901
	Arcuate_Fasciculus_R	0.879
	Arcuate_Fasciculus_L	0.868
	Superior_Longitudinal_Fasciculus_1_L	0.857
	Corticospinal_Tract_L	0.835
	Middle_Cerebellar_Peduncle	0.835
	Cingulum_subsection_Peri_genual_R	0.824
	Superior_Longitudinal_Fasciculus_2_L	0.824
	Superior_Longitudinal_Fasciculus_3_R	0.824
	Uncinate_Fasciculus_L	0.824
	Forceps_Major	0.791
	Superior_Longitudinal_Fasciculus_2_R	0.791
	Superior_Longitudinal_Fasciculus_3_L	0.791
	Superior_Thalamic_Radiation_R	0.769
	Cingulum_subsection_Dorsal_R	0.758
	Corticospinal_Tract_R	0.758
	Frontal_Aslant_Tract_R	0.758
	Vertical_Occipital_Fasciculus_R	0.758
	Anterior_Thalamic_Radiation_L	0.725
	Superior_Thalamic_Radiation_L	0.725
	Anterior_Thalamic_Radiation_R	0.714
	Cingulum_subsection_Temporal_R	0.714
	Fornix_L	0.714
KFA		
	Middle_Longitudinal_Fasciculus_R	0.890
	Middle_Cerebellar_Peduncle	0.868
	Cingulum_subsection_Peri_genual_L	0.780
	Inferior_Fronto_Occipital_Fasciculus_R	0.769
	Inferior_Longitudinal_Fasciculus_L	0.758
	Superior_Thalamic_Radiation_L	0.758
	Superior_Longitudinal_Fasciculus_2_R	0.747
	Cingulum_subsection_Peri_genual_R	0.736
	Cingulum_subsection_Dorsal_L	0.725
	Vertical_Occipital_Fasciculus_R	0.703
MK		
	Cingulum_subsection_Peri_genual_R	0.879
	Anterior_Commissure	0.780
	Anterior_Thalamic_Radiation_L	0.758
	Superior_Thalamic_Radiation_L	0.725
RK		
	Forceps_Minor	0.824
	Superior_Longitudinal_Fasciculus_2_R	0.802
	Arcuate_Fasciculus_R	0.747

	Superior_Longitudinal_Fasciculus_3_R	0.747
	Anterior_Thalamic_Radiation_R	0.736
	Forceps_Major	0.736
FA		
	Frontal_Aslant_Tract_L	0.890
	Inferior_Fronto_Occipital_Fasciculus_R	0.890
	Uncinate_Fasciculus_R	0.835
	Arcuate_Fasciculus_L	0.824
	Vertical_Occipital_Fasciculus_L	0.824
	Forceps_Major	0.813
	Superior_Longitudinal_Fasciculus_2_L	0.791
	Superior_Longitudinal_Fasciculus_3_L	0.791
	Cingulum_subsection_Peri_genual_R	0.780
	Cingulum_subsection_Dorsal_R	0.780
	Optic_Radiation_R	0.769
	Superior_Longitudinal_Fasciculus_1_L	0.758
	Vertical_Occipital_Fasciculus_R	0.758
	Middle_Cerebellar_Peduncle	0.747
	Superior_Longitudinal_Fasciculus_2_R	0.736
	Corticospinal_Tract_L	0.725
	Anterior_Thalamic_Radiation_R	0.714
NG		
	Inferior_Longitudinal_Fasciculus_L	0.813
NGRad		
	Inferior_Longitudinal_Fasciculus_L	0.736
RTAP		
	Inferior_Fronto_Occipital_Fasciculus_R	0.964
	Anterior_Thalamic_Radiation_R	0.954
	Arcuate_Fasciculus_L	0.923
	Middle_Longitudinal_Fasciculus_L	0.912
	Corticospinal_Tract_L	0.890
	Inferior_Longitudinal_Fasciculus_L	0.890
	Superior_Longitudinal_Fasciculus_3_R	0.890
	Arcuate_Fasciculus_R	0.879
	Superior_Longitudinal_Fasciculus_2_R	0.879
	Superior_Longitudinal_Fasciculus_3_L	0.879
	Uncinate_Fasciculus_R	0.879
	Anterior_Thalamic_Radiation_L	0.868
	Forceps_Minor	0.868
	Uncinate_Fasciculus_L	0.857
	Vertical_Occipital_Fasciculus_R	0.857
	Frontal_Aslant_Tract_R	0.846
	Inferior_Fronto_Occipital_Fasciculus_L	0.846
	Optic_Radiation_R	0.835
	Superior_Longitudinal_Fasciculus_1_L	0.835
	Corticospinal_Tract_R	0.824
	Forceps_Major	0.824
	Middle_Longitudinal_Fasciculus_R	0.824
	Superior_Longitudinal_Fasciculus_2_L	0.824
	Vertical_Occipital_Fasciculus_L	0.824
	Frontal_Aslant_Tract_L	0.813

	Acoustic_Radiation_L	0.802
	Cingulum_subsection_Peri_genual_L	0.802
	Superior_Thalamic_Radiation_L	0.780
	Optic_Radiation_L	0.769
	Superior_Longitudinal_Fasciculus_1_R	0.758
	Inferior_Longitudinal_Fasciculus_R	0.747
	Superior_Thalamic_Radiation_R	0.736
	Cingulum_subsection_Peri_genual_R	0.714
	Cingulum_subsection_Dorsal_R	0.703
RTOP		
	Inferior_Fronto_Occipital_Fasciculus_R	0.934
	Corticospinal_Tract_L	0.923
	Arcuate_Fasciculus_L	0.912
	Anterior_Thalamic_Radiation_R	0.890
	Frontal_Aslant_Tract_R	0.868
	Superior_Longitudinal_Fasciculus_3_L	0.868
	Superior_Longitudinal_Fasciculus_3_R	0.857
	Anterior_Thalamic_Radiation_L	0.846
	Cingulum_subsection_Peri_genual_R	0.846
	Frontal_Aslant_Tract_L	0.846
	Vertical_Occipital_Fasciculus_L	0.846
	Cingulum_subsection_Peri_genual_L	0.835
	Inferior_Fronto_Occipital_Fasciculus_L	0.835
	Superior_Longitudinal_Fasciculus_2_L	0.835
	Middle_Longitudinal_Fasciculus_L	0.824
	Optic_Radiation_L	0.824
	Superior_Longitudinal_Fasciculus_1_L	0.824
	Forceps_Major	0.813
	Forceps_Minor	0.813
	Inferior_Longitudinal_Fasciculus_L	0.813
	Superior_Thalamic_Radiation_L	0.791
	Uncinate_Fasciculus_L	0.791
	Acoustic_Radiation_L	0.769
	Inferior_Longitudinal_Fasciculus_R	0.769
	Arcuate_Fasciculus_R	0.758
	Superior_Longitudinal_Fasciculus_1_R	0.758
	Uncinate_Fasciculus_R	0.758
	Anterior_Commissure	0.747
	Superior_Longitudinal_Fasciculus_2_R	0.736
	Cingulum_subsection_Dorsal_L	0.714
	Superior_Thalamic_Radiation_R	0.714
	Vertical_Occipital_Fasciculus_R	0.714
ICVF		
	Corticospinal_Tract_L	0.879
	Superior_Longitudinal_Fasciculus_1_L	0.857
	Forceps_Major	0.802
	Arcuate_Fasciculus_L	0.758

Additional Table 2 B The AUC of White Matter Fiber Tract Metrics Between HC and NPSLE

Metrics	Brain region	AUC
AK		
	Superior_Thalamic_Radiation_L	0.930
	Fornix_R	0.916
	Anterior_Commissure	0.909
	Inferior_Fronto_Occipital_Fasciculus_L	0.909
	Middle_Longitudinal_Fasciculus_L	0.902
	Optic_Radiation_L	0.902
	Anterior_Thalamic_Radiation_R	0.895
	Arcuate_Fasciculus_R	0.895
	Superior_Longitudinal_Fasciculus_2_R	0.874
	Superior_Longitudinal_Fasciculus_1_L	0.867
	Superior_Longitudinal_Fasciculus_2_L	0.867
	Arcuate_Fasciculus_L	0.853
	Corticospinal_Tract_L	0.853
	Anterior_Thalamic_Radiation_L	0.839
	Cingulum_subsection_Peri_genual_R	0.839
	Frontal_Aslant_Tract_L	0.839
	Frontal_Aslant_Tract_R	0.832
	Superior_Longitudinal_Fasciculus_3_R	0.832
	Inferior_Fronto_Occipital_Fasciculus_R	0.825
	Superior_Longitudinal_Fasciculus_3_L	0.825
	Superior_Thalamic_Radiation_R	0.825
	Cingulum_subsection_Temporal_R	0.818
	Acoustic_Radiation_L	0.811
	Cingulum_subsection_Peri_genual_L	0.811
	Fornix_L	0.804
	Forceps_Major	0.783
	Inferior_Longitudinal_Fasciculus_L	0.783
	Optic_Radiation_R	0.776
	Forceps_Minor	0.748
	Middle_Cerebellar_Peduncle	0.748
	Vertical_Occipital_Fasciculus_L	0.748
	Corticospinal_Tract_R	0.741
	Uncinate_Fasciculus_L	0.741
GA		
	Forceps_Minor	0.972
	Vertical_Occipital_Fasciculus_L	0.958
	Uncinate_Fasciculus_R	0.937
	Cingulum_subsection_Peri_genual_L	0.916
	Anterior_Thalamic_Radiation_R	0.909
	Cingulum_subsection_Dorsal_L	0.902
	Cingulum_subsection_Dorsal_R	0.902
	Inferior_Fronto_Occipital_Fasciculus_L	0.902
	Cingulum_subsection_Peri_genual_R	0.895
	Superior_Longitudinal_Fasciculus_1_L	0.895
	Uncinate_Fasciculus_L	0.895
	Forceps_Major	0.888
	Anterior_Thalamic_Radiation_L	0.881
	Superior_Longitudinal_Fasciculus_3_L	0.881
	Fornix_L	0.874

	Arcuate_Fasciculus_L	0.867
	Inferior_Fronto_Occipital_Fasciculus_R	0.867
	Superior_Longitudinal_Fasciculus_3_R	0.867
	Anterior_Commissure	0.853
	Middle_Longitudinal_Fasciculus_L	0.853
	Middle_Cerebellar_Peduncle	0.846
	Superior_Longitudinal_Fasciculus_1_R	0.846
	Fornix_R	0.839
	Optic_Radiation_L	0.839
	Superior_Longitudinal_Fasciculus_2_R	0.839
	Inferior_Longitudinal_Fasciculus_L	0.818
	Superior_Longitudinal_Fasciculus_2_L	0.811
	Vertical_Occipital_Fasciculus_R	0.811
	Arcuate_Fasciculus_R	0.797
	Corticospinal_Tract_L	0.790
	Corticospinal_Tract_R	0.790
	Frontal_Aslant_Tract_L	0.790
	Frontal_Aslant_Tract_R	0.790
	Inferior_Longitudinal_Fasciculus_R	0.790
	Superior_Thalamic_Radiation_R	0.776
	Optic_Radiation_R	0.769
	Cingulum_subsection_Temporal_R	0.762
	Superior_Thalamic_Radiation_L	0.762
	Middle_Longitudinal_Fasciculus_R	0.755
	Acoustic_Radiation_L	0.741
KFA		
	Anterior_Thalamic_Radiation_L	0.923
	Middle_Cerebellar_Peduncle	0.923
	Anterior_Commissure	0.916
	Fornix_L	0.916
	Inferior_Fronto_Occipital_Fasciculus_R	0.916
	Superior_Longitudinal_Fasciculus_1_L	0.916
	Forceps_Minor	0.909
	Uncinate_Fasciculus_R	0.909
	Cingulum_subsection_Peri_genual_L	0.902
	Superior_Longitudinal_Fasciculus_2_R	0.895
	Fornix_R	0.881
	Superior_Longitudinal_Fasciculus_3_L	0.881
	Superior_Longitudinal_Fasciculus_3_R	0.881
	Anterior_Thalamic_Radiation_R	0.874
	Cingulum_subsection_Peri_genual_R	0.874
	Superior_Thalamic_Radiation_L	0.874
	Corticospinal_Tract_R	0.867
	Vertical_Occipital_Fasciculus_L	0.867
	Cingulum_subsection_Dorsal_R	0.860
	Superior_Longitudinal_Fasciculus_1_R	0.860
	Forceps_Major	0.853
	Frontal_Aslant_Tract_L	0.853
	Cingulum_subsection_Dorsal_L	0.846
	Inferior_Fronto_Occipital_Fasciculus_L	0.846
	Acoustic_Radiation_L	0.839

	Arcuate_Fasciculus_L	0.839
	Uncinate_Fasciculus_L	0.839
	Middle_Longitudinal_Fasciculus_L	0.832
	Optic_Radiation_L	0.825
	Inferior_Longitudinal_Fasciculus_R	0.811
	Superior_Longitudinal_Fasciculus_2_L	0.804
	Arcuate_Fasciculus_R	0.797
	Frontal_Aslant_Tract_R	0.797
	Superior_Thalamic_Radiation_R	0.797
	Optic_Radiation_R	0.790
	Vertical_Occipital_Fasciculus_R	0.783
	Middle_Longitudinal_Fasciculus_R	0.776
	Corticospinal_Tract_L	0.769
	Inferior_Longitudinal_Fasciculus_L	0.762
	Acoustic_Radiation_R	0.713
MK		
	Fornix_R	0.909
	Arcuate_Fasciculus_L	0.902
	Acoustic_Radiation_L	0.895
	Middle_Longitudinal_Fasciculus_L	0.888
	Corticospinal_Tract_L	0.881
	Optic_Radiation_L	0.881
	Superior_Longitudinal_Fasciculus_3_R	0.881
	Anterior_Thalamic_Radiation_R	0.860
	Cingulum_subsection_Peri_genual_R	0.860
	Superior_Longitudinal_Fasciculus_3_L	0.860
	Cingulum_subsection_Dorsal_R	0.853
	Forceps_Major	0.853
	Inferior_Longitudinal_Fasciculus_L	0.853
	Superior_Longitudinal_Fasciculus_2_L	0.853
	Vertical_Occipital_Fasciculus_L	0.853
	Forceps_Minor	0.846
	Frontal_Aslant_Tract_R	0.846
	Inferior_Fronto_Occipital_Fasciculus_L	0.846
	Arcuate_Fasciculus_R	0.839
	Cingulum_subsection_Peri_genual_L	0.839
	Fornix_L	0.839
	Superior_Longitudinal_Fasciculus_2_R	0.839
	Superior_Longitudinal_Fasciculus_1_L	0.832
	Anterior_Commissure	0.825
	Superior_Longitudinal_Fasciculus_1_R	0.825
	Cingulum_subsection_Temporal_R	0.804
	Superior_Thalamic_Radiation_R	0.804
	Inferior_Fronto_Occipital_Fasciculus_R	0.790
	Corticospinal_Tract_R	0.783
	Superior_Thalamic_Radiation_L	0.783
	Frontal_Aslant_Tract_L	0.776
	Uncinate_Fasciculus_L	0.776
	Uncinate_Fasciculus_R	0.776
	Vertical_Occipital_Fasciculus_R	0.776
	Anterior_Thalamic_Radiation_L	0.769

Inferior_Longitudinal_Fasciculus_R	0.769
Acoustic_Radiation_R	0.762
Middle_Longitudinal_Fasciculus_R	0.755
Optic_Radiation_R	0.755
Cingulum_subsection_Temporal_L	0.720

RK

Arcuate_Fasciculus_L	0.923
Anterior_Thalamic_Radiation_R	0.909
Superior_Longitudinal_Fasciculus_3_R	0.909
Arcuate_Fasciculus_R	0.902
Superior_Longitudinal_Fasciculus_3_L	0.902
Cingulum_subsection_Peri_genual_L	0.888
Fornix_R	0.888
Forceps_Minor	0.881
Fornix_L	0.874
Inferior_Longitudinal_Fasciculus_L	0.874
Superior_Longitudinal_Fasciculus_2_L	0.874
Uncinate_Fasciculus_L	0.874
Uncinate_Fasciculus_R	0.867
Cingulum_subsection_Peri_genual_R	0.860
Vertical_Occipital_Fasciculus_L	0.860
Inferior_Fronto_Occipital_Fasciculus_L	0.853
Inferior_Fronto_Occipital_Fasciculus_R	0.853
Middle_Longitudinal_Fasciculus_L	0.853
Optic_Radiation_L	0.853
Superior_Longitudinal_Fasciculus_2_R	0.853
Forceps_Major	0.846
Superior_Longitudinal_Fasciculus_1_L	0.846
Acoustic_Radiation_L	0.832
Anterior_Thalamic_Radiation_L	0.832
Corticospinal_Tract_L	0.832
Acoustic_Radiation_R	0.818
Frontal_Aslant_Tract_R	0.811
Frontal_Aslant_Tract_L	0.804
Superior_Thalamic_Radiation_L	0.804
Superior_Thalamic_Radiation_R	0.804
Corticospinal_Tract_R	0.790
Inferior_Longitudinal_Fasciculus_R	0.790
Optic_Radiation_R	0.790
Superior_Longitudinal_Fasciculus_1_R	0.790
Vertical_Occipital_Fasciculus_R	0.790
Middle_Longitudinal_Fasciculus_R	0.783
Anterior_Commissure	0.769
Cingulum_subsection_Dorsal_R	0.755

FA

Forceps_Minor	0.965
Vertical_Occipital_Fasciculus_L	0.951
Uncinate_Fasciculus_R	0.937
Inferior_Fronto_Occipital_Fasciculus_R	0.930
Anterior_Thalamic_Radiation_R	0.923
Superior_Longitudinal_Fasciculus_3_L	0.923

Cingulum_subsection_Peri_genual_L	0.916
Superior_Longitudinal_Fasciculus_3_R	0.916
Uncinate_Fasciculus_L	0.916
Cingulum_subsection_Dorsal_R	0.909
Middle_Cerebellar_Peduncle	0.909
Arcuate_Fasciculus_L	0.902
Forceps_Major	0.895
Cingulum_subsection_Dorsal_L	0.881
Superior_Longitudinal_Fasciculus_2_R	0.881
Inferior_Fronto_Occipital_Fasciculus_L	0.874
Middle_Longitudinal_Fasciculus_L	0.867
Anterior_Thalamic_Radiation_L	0.846
Cingulum_subsection_Peri_genual_R	0.846
Inferior_Longitudinal_Fasciculus_L	0.846
Corticospinal_Tract_R	0.839
Fornix_L	0.839
Inferior_Longitudinal_Fasciculus_R	0.839
Superior_Longitudinal_Fasciculus_2_L	0.839
Vertical_Occipital_Fasciculus_R	0.839
Optic_Radiation_L	0.832
Superior_Thalamic_Radiation_R	0.832
Superior_Longitudinal_Fasciculus_1_L	0.825
Superior_Longitudinal_Fasciculus_1_R	0.825
Arcuate_Fasciculus_R	0.818
Frontal_Aslant_Tract_R	0.818
Fornix_R	0.811
Anterior_Commissure	0.804
Superior_Thalamic_Radiation_L	0.804
Frontal_Aslant_Tract_L	0.797
Middle_Longitudinal_Fasciculus_R	0.790
Acoustic_Radiation_R	0.776
Optic_Radiation_R	0.769
Cingulum_subsection_Temporal_R	0.762
Corticospinal_Tract_L	0.762
Acoustic_Radiation_L	0.727

RD

Fornix_R	0.888
Anterior_Commissure	0.832
Forceps_Minor	0.818
Fornix_L	0.818
Superior_Longitudinal_Fasciculus_3_R	0.818
Superior_Longitudinal_Fasciculus_3_L	0.811
Superior_Longitudinal_Fasciculus_2_L	0.797
Uncinate_Fasciculus_R	0.797
Cingulum_subsection_Dorsal_R	0.790
Forceps_Major	0.783
Inferior_Fronto_Occipital_Fasciculus_R	0.783
Anterior_Thalamic_Radiation_L	0.776
Anterior_Thalamic_Radiation_R	0.776
Vertical_Occipital_Fasciculus_L	0.776
Cingulum_subsection_Peri_genual_R	0.769

	Arcuate_Fasciculus_L	0.762
	Inferior_Fronto_Occipital_Fasciculus_L	0.762
	Arcuate_Fasciculus_R	0.755
	Uncinate_Fasciculus_L	0.755
	Frontal_Aslant_Tract_R	0.748
	Optic_Radiation_L	0.748
	Middle_Longitudinal_Fasciculus_R	0.741
	Vertical_Occipital_Fasciculus_R	0.741
	Cingulum_subsection_Peri_genual_L	0.734
	Frontal_Aslant_Tract_L	0.734
	Middle_Longitudinal_Fasciculus_L	0.727
	Optic_Radiation_R	0.727
	Inferior_Longitudinal_Fasciculus_R	0.713
	Cingulum_subsection_Dorsal_L	0.706
	Corticospinal_Tract_R	0.706
MSD		
	Fornix_R	0.895
	Superior_Thalamic_Radiation_R	0.853
	Cingulum_subsection_Peri_genual_R	0.839
	Corticospinal_Tract_R	0.818
	Fornix_L	0.811
	Anterior_Commissure	0.804
	Acoustic_Radiation_L	0.797
	Superior_Thalamic_Radiation_L	0.776
	Cingulum_subsection_Peri_genual_L	0.769
	Optic_Radiation_R	0.755
	Anterior_Thalamic_Radiation_L	0.748
	Forceps_Major	0.748
	Superior_Longitudinal_Fasciculus_3_R	0.741
	Anterior_Thalamic_Radiation_R	0.727
	Acoustic_Radiation_R	0.720
	Frontal_Aslant_Tract_R	0.720
	Arcuate_Fasciculus_R	0.713
	Superior_Longitudinal_Fasciculus_2_R	0.713
	Inferior_Fronto_Occipital_Fasciculus_R	0.706
NG		
	Cingulum_subsection_Peri_genual_R	0.706
	Cingulum_subsection_Dorsal_R	0.706
RTAP		
	Vertical_Occipital_Fasciculus_L	0.937
	Forceps_Minor	0.930
	Fornix_R	0.930
	Anterior_Thalamic_Radiation_R	0.916
	Fornix_L	0.909
	Superior_Longitudinal_Fasciculus_3_R	0.909
	Arcuate_Fasciculus_L	0.895
	Cingulum_subsection_Dorsal_R	0.895
	Cingulum_subsection_Peri_genual_R	0.888
	Superior_Longitudinal_Fasciculus_3_L	0.881
	Cingulum_subsection_Peri_genual_L	0.874
	Forceps_Major	0.867

	Uncinate_Fasciculus_R	0.867
	Arcuate_Fasciculus_R	0.860
	Middle_Longitudinal_Fasciculus_L	0.860
	Superior_Longitudinal_Fasciculus_2_R	0.860
	Inferior_Fronto_Occipital_Fasciculus_L	0.853
	Inferior_Fronto_Occipital_Fasciculus_R	0.846
	Middle_Cerebellar_Peduncle	0.846
	Uncinate_Fasciculus_L	0.846
	Anterior_Thalamic_Radiation_L	0.839
	Superior_Longitudinal_Fasciculus_2_L	0.839
	Acoustic_Radiation_L	0.832
	Frontal_Aslant_Tract_R	0.832
	Inferior_Longitudinal_Fasciculus_L	0.832
	Optic_Radiation_L	0.832
	Superior_Longitudinal_Fasciculus_1_L	0.832
	Cingulum_subsection_Dorsal_L	0.818
	Frontal_Aslant_Tract_L	0.818
	Vertical_Occipital_Fasciculus_R	0.818
	Anterior_Commissure	0.804
	Corticospinal_Tract_L	0.804
	Inferior_Longitudinal_Fasciculus_R	0.790
	Superior_Longitudinal_Fasciculus_1_R	0.783
	Superior_Thalamic_Radiation_L	0.783
	Corticospinal_Tract_R	0.776
	Superior_Thalamic_Radiation_R	0.776
	Optic_Radiation_R	0.762
	Middle_Longitudinal_Fasciculus_R	0.755
	Cingulum_subsection_Temporal_R	0.748
	Acoustic_Radiation_R	0.713
RTOP		
	Fornix_L	0.930
	Fornix_R	0.930
	Vertical_Occipital_Fasciculus_L	0.909
	Middle_Longitudinal_Fasciculus_L	0.874
	Cingulum_subsection_Peri_genual_R	0.867
	Forceps_Minor	0.867
	Superior_Longitudinal_Fasciculus_2_R	0.867
	Arcuate_Fasciculus_L	0.860
	Cingulum_subsection_Dorsal_R	0.860
	Acoustic_Radiation_L	0.853
	Anterior_Thalamic_Radiation_R	0.853
	Superior_Longitudinal_Fasciculus_3_L	0.853
	Cingulum_subsection_Peri_genual_L	0.846
	Inferior_Fronto_Occipital_Fasciculus_L	0.846
	Optic_Radiation_L	0.846
	Superior_Longitudinal_Fasciculus_3_R	0.846
	Forceps_Major	0.839
	Frontal_Aslant_Tract_R	0.839
	Inferior_Longitudinal_Fasciculus_L	0.839
	Middle_Cerebellar_Peduncle	0.839
	Anterior_Thalamic_Radiation_L	0.825

	Arcuate_Fasciculus_R	0.825
	Superior_Longitudinal_Fasciculus_2_L	0.825
	Superior_Longitudinal_Fasciculus_1_L	0.818
	Uncinate_Fasciculus_R	0.818
	Anterior_Commissure	0.811
	Corticospinal_Tract_L	0.811
	Frontal_Aslant_Tract_L	0.811
	Uncinate_Fasciculus_L	0.811
	Inferior_Fronto_Occipital_Fasciculus_R	0.808
	Cingulum_subsection_Temporal_R	0.797
	Vertical_Occipital_Fasciculus_R	0.797
	Corticospinal_Tract_R	0.783
	Inferior_Longitudinal_Fasciculus_R	0.776
	Superior_Longitudinal_Fasciculus_1_R	0.776
	Cingulum_subsection_Dorsal_L	0.755
	Optic_Radiation_R	0.755
	Superior_Thalamic_Radiation_R	0.741
	Middle_Longitudinal_Fasciculus_R	0.734
	Superior_Thalamic_Radiation_L	0.734
	Acoustic_Radiation_R	0.706
ECVF		
	Superior_Longitudinal_Fasciculus_3_R	0.888
	Arcuate_Fasciculus_L	0.881
	Arcuate_Fasciculus_R	0.874
	Acoustic_Radiation_L	0.853
	Forceps_Minor	0.846
	Superior_Longitudinal_Fasciculus_1_L	0.846
	Superior_Longitudinal_Fasciculus_3_L	0.846
	Cingulum_subsection_Peri_genual_R	0.839
	Cingulum_subsection_Dorsal_R	0.839
	Middle_Longitudinal_Fasciculus_L	0.839
	Superior_Longitudinal_Fasciculus_2_R	0.839
	Anterior_Thalamic_Radiation_R	0.832
	Corticospinal_Tract_L	0.832
	Inferior_Fronto_Occipital_Fasciculus_L	0.832
	Optic_Radiation_L	0.832
	Superior_Longitudinal_Fasciculus_2_L	0.832
	Vertical_Occipital_Fasciculus_R	0.825
	Frontal_Aslant_Tract_R	0.818
	Uncinate_Fasciculus_L	0.811
	Inferior_Fronto_Occipital_Fasciculus_R	0.804
	Inferior_Longitudinal_Fasciculus_L	0.804
	Vertical_Occipital_Fasciculus_L	0.804
	Forceps_Major	0.797
	Anterior_Thalamic_Radiation_L	0.790
	Cingulum_subsection_Peri_genual_L	0.783
	Inferior_Longitudinal_Fasciculus_R	0.783
	Cingulum_subsection_Temporal_R	0.755
	Superior_Longitudinal_Fasciculus_1_R	0.755
	Superior_Thalamic_Radiation_R	0.748
	Cingulum_subsection_Dorsal_L	0.741

	Frontal_Aslant_Tract_L	0.741
	Optic_Radiation_R	0.741
	Superior_Thalamic_Radiation_L	0.741
	Corticospinal_Tract_R	0.734
	Anterior_Commissure	0.720
	Uncinate_Fasciculus_R	0.720
	Middle_Longitudinal_Fasciculus_R	0.713
ICVF		
	Vertical_Occipital_Fasciculus_L	0.895
	Cingulum_subsection_Dorsal_R	0.888
	Middle_Longitudinal_Fasciculus_L	0.881
	Cingulum_subsection_Peri_genual_R	0.874
	Superior_Longitudinal_Fasciculus_3_R	0.874
	Arcuate_Fasciculus_L	0.860
	Acoustic_Radiation_L	0.846
	Forceps_Minor	0.846
	Superior_Longitudinal_Fasciculus_2_L	0.846
	Forceps_Major	0.839
	Frontal_Aslant_Tract_R	0.839
	Superior_Longitudinal_Fasciculus_2_R	0.839
	Optic_Radiation_L	0.832
	Arcuate_Fasciculus_R	0.825
	Cingulum_subsection_Peri_genual_L	0.825
	Superior_Longitudinal_Fasciculus_1_L	0.825
	Superior_Longitudinal_Fasciculus_3_L	0.825
	Anterior_Thalamic_Radiation_R	0.811
	Corticospinal_Tract_L	0.811
	Inferior_Fronto_Occipital_Fasciculus_L	0.811
	Inferior_Longitudinal_Fasciculus_L	0.790
	Superior_Longitudinal_Fasciculus_1_R	0.790
	Uncinate_Fasciculus_R	0.790
	Cingulum_subsection_Temporal_R	0.783
	Vertical_Occipital_Fasciculus_R	0.783
	Cingulum_subsection_Dorsal_L	0.769
	Inferior_Fronto_Occipital_Fasciculus_R	0.769
	Anterior_Thalamic_Radiation_L	0.762
	Corticospinal_Tract_R	0.762
	Uncinate_Fasciculus_L	0.755
	Frontal_Aslant_Tract_L	0.741
	Inferior_Longitudinal_Fasciculus_R	0.741
	Middle_Cerebellar_Peduncle	0.741
	Superior_Thalamic_Radiation_R	0.741
	Fornix_L	0.734
	Middle_Longitudinal_Fasciculus_R	0.734
	Anterior_Commissure	0.727
	Optic_Radiation_R	0.727
	Superior_Thalamic_Radiation_L	0.727
